# Neutral vs Charged Luminescent Radicals: Anti-Kasha Emission and the Impact of Molecular Surrounding

**DOI:** 10.1021/acs.jpca.4c02779

**Published:** 2024-06-20

**Authors:** I. Sahalianov, R. R. Valiev, R. R. Ramazanov, G. Baryshnikov

**Affiliations:** † Laboratory of Organic Electronics, Department of Science and Technology, Linköping University, SE-60174 Norrköping, Sweden; ‡ Wallenberg Initiative Materials Science for Sustainability, ITN, Linköping University, 60174 Norrköping,Sweden; § Department of Chemistry, University of Helsinki, P.O. Box 55 (A.I. Virtanens plats 1), 00014Helsinki,Finland

## Abstract

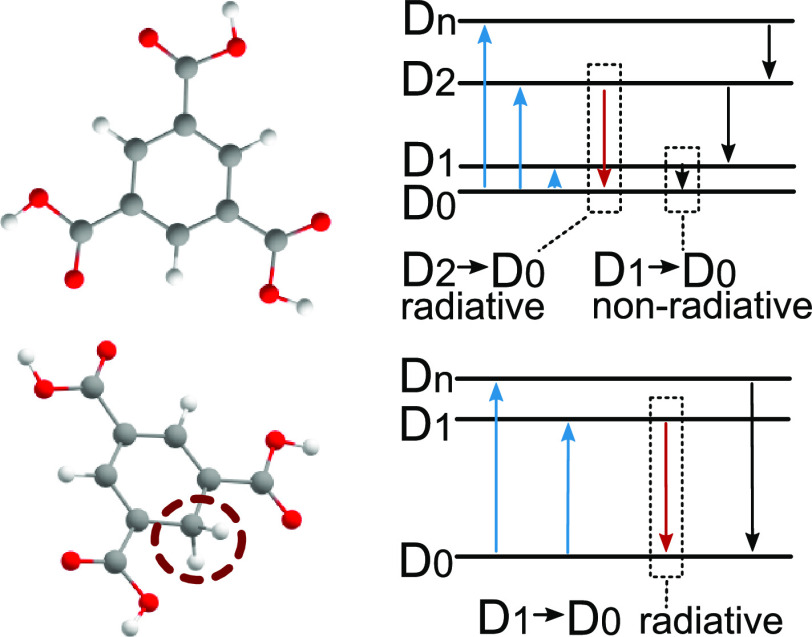

Organic luminescent materials attract growing interest as an elegant solution for sustainable and inexpensive light-emitting devices. Most of them are neutral-emitting molecules with an implicit restriction of 25% internal quantum efficiency due to a spin-forbidden nature of the T_1_ → S_0_ transition. Utilizing organic radicals allows one to overcome such limits by theoretically boosting quantum yield up to 100%. Recently, different light-emitting radicals based on carbonyl- and carboxyl-substituted benzenes were synthesized and stabilized in different polymer matrices or ionic liquids. While some of them were proved to be suitable luminescent materials, the exact theoretical explanation of the nature of their emission is missing. There are two main hypotheses proposed in the literature. The first one suggests that the origin of luminescence is D_2_ → D_0_ anti-Kasha emission from anion radicals, while the second theory is based on D_1_ → D_0_ Kasha emission from neutral protonated radicals. In this work, we investigate both hypotheses and compare their derivatives with the available experimental data. We used density functional theory and complete-active space perturbation theory to investigate the absorption and emission properties in various aromatic carbonyl radicals. We found that both emission mechanisms can coexist simultaneously, with a dominant emission contribution made by anion radicals because of better agreement between oscillator strengths and radiative rate constants. Our numerical simulations agree with the experimental data and provide theoretical foundations for the fabrication of next-generation light-emitting devices based on luminescent radicals.

## Introduction

Modern industry standards demand materials that are efficient not only in performance but also in sustainability. Light-emitting materials are not an exception in this case. Current inorganic light-emitting diodes have been in active development for decades, resulting in technological solutions that balance materials price and production costs.^1^ Organic materials have several benefits compared to their inorganic competitors, the most important of which are ease of fabrication, superior mechanics properties, and overall sustainability.^2^ While organic light-emitting diodes (OLEDs) are currently expensive in fabrication, potential technological development can overcome this problem and make OLEDs a sustainable and abundant alternative to inorganic analogs.^3^ However, organic emitters can have another advantage, which puts them far above inorganic ones. Their emission efficiency is not limited by 25%. Luminescent radicals exhibit an increased interest among organic emitters because of potentially 100% quantum yield.^4^ While neutral singlet molecules are losing up to 75% of their efficiency because of S_*n*_ → T_1_ intersystem crossing, an emission in luminescent radicals originates from the transition from excited doublet state D_*n*_ to doublet ground state D_0_. The only restrictions on emission efficiency are caused by the symmetry-forbidden transitions and engineering limitations originating from the exact LED architecture.

Several different compounds were investigated as prominent light-emitting radicals. The cornerstone of organic radicals triphenylmethyl (TM) was synthesized by Gomberg in 1900,^5^ followed by its more stable version and tris (2,4,6-trichlorophenyl)methane (TTM)^6^ and perchlorotriphenyl methyl (PTM).^7^ In recent years, other variations of these compounds such as PS-CzTTM,^8^ TTM-3NCz,^9^ CzBTM,^10^ and others have been used to fabricate various OLEDs^4^ successfully. Stabilization of radicals can be done by using polymer matrix poly(vinyl alcohol) (PVA),^11^ poly(methyl methacrylate) (PMMA),^12^ polystyrene (PS),^13^ polyvinylpyrrolidone (PVP), or solvation in ionic liquids.^14^

Anti-Kasha emission is a phenomenon, usually referred to as the delayed fluorescence in neutral molecules caused by the radiative transitions from higher excited states.^15^ Kasha’s rule suggests that emission should closely follow an excitation within nanoseconds, with the transition from the lowest excited state of a given multiplicity (S_1_ or T_1_) into the ground state.^16^ In some compounds, emission can happen with the transition from higher excited states S_*N*_ → S_0_, which often occurs at longer times compared to the S_1_ → S_0_ transition. Possible compounds, demonstrating this kind of emission include various azulene derivatives, metal complexes, etc.^17−20^ Anti-Kasha emission can be classified into three categories: the strong electronic weak vibrational nonadiabatic coupling (NAC) regime (type I), the strong electronic strong vibrational NAC regime (type II), and the very weak electronic NAC regime.^21^ Type I emission regime implies a large energy gap between S_2_ and S_1_ states, resulting in slowing down internal conversion and stimulation of S_2_ → S_0_ radiative transition.^22^ Type II emission regime implies a small gap between S_2_–S_1_ (close to kT) with a spontaneous repopulation of S_2_ from S_1_ and a high probability of S_2_ → S_0_ emissive transition.^20^ Finally, the III regime unites compounds with a small S_2_–S_1_ gap but negligibly low internal conversion due to the lack of electron-vibrational coupling.^21^

A variety of observed anti-Kasha emission regimes comes with an increasingly larger number of systems, reported to violate the Kasha rule. The complexity of the emission processes in some systems may even lead to incorrect classification when a compound that seemingly violates the Kasha rule happens to demonstrate other emission mechanisms according to the Kasha rule.^23^ Possible sources of emission that are misinterpreted as anti-Kasha can originate from UV-stimulated isomerization of emitting molecules,^24^ the formation of tautomers^25^ or rotamers. Before excitation, UV irradiation may stimulate proton-coupled electron transfer (PCET) through different pathways.^26^ Alternatively, after the excitation of molecules, emission can be affected by excited-state intramolecular proton transfer (ESIPT).^27^

**Figure 1:**
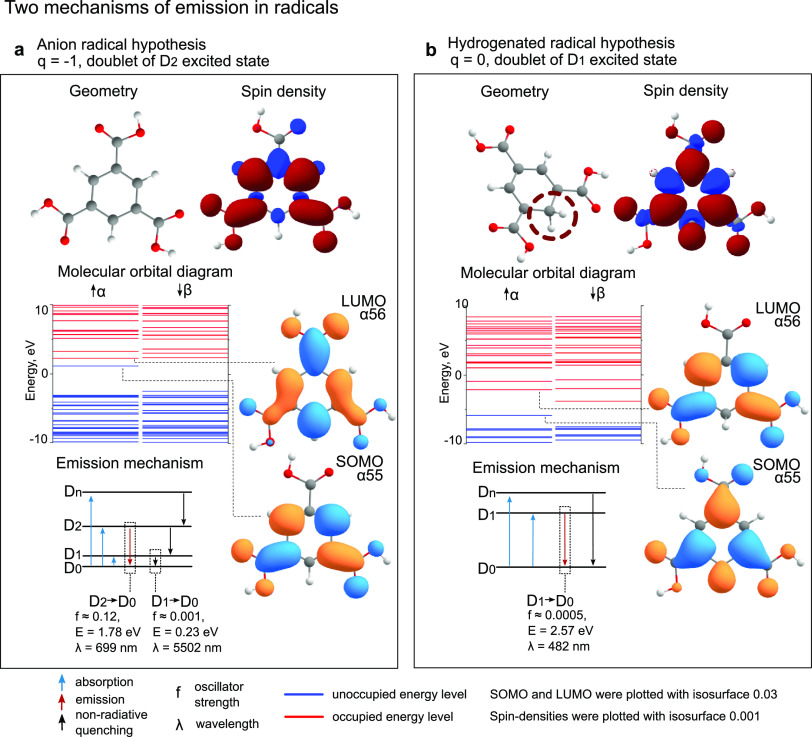
Summary of the difference between the hypothesis of anion (a) and hydrogenated (b) radical emission on an example of Ph-3COOH. The protonated region is highlighted by a dashed circle. The energy diagram data were calculated with B3LYP/6-31G(d)/TDA while the emission diagram was calculated at ωB97XD/6-31G(d)/TDA.

Inspired by TM, benzoic acids were modified with carbonyl electron withdroved groups, resulting in several *n*-substituted benzenes (*n* = 1–6). They were experimentally verified to be stable in different polymer matrixes, with their emission properties highly dependent on the number of substituting groups.^14,26,28^ Despite the extensive experimental studies of such compounds, the origins of the emission remain under debate. The geometry of these compounds is too simplistic for UV-stimulated isomerization or the formation of tautomers. ESIPT or PCET in different ways is hindered by the difficulty in protonation of a benzene ring, the probability of which is rather questionable. As a possible explanation, two different emission mechanisms were proposed. The first is based on the assumption that doublets originate from charge transfer and the formation of emissive anions. Anions can be formed through photoinduced charge transfer between dopant molecules, resulting in the formation of anion + cation pair, or directly through photoinduced electron transfer from the polymer matrix,^29^ with further stabilization of radicals by H-bond surroundings. In case an anion + cation pair is formed, anions are emissive. At the same time, cations or neutral counterparts usually do not exhibit any luminescent capabilities in visible region^28,30^ (see Tables S1 and S2, except cationic emission reported in the study by Tong et al.^31^). In such anionic compounds, the first exited doublet state (D_1_) is negligibly close to the ground state of the molecules, making the D_1_ → D_0_ energy gap so narrow (<0.5 eV) that it prevents emissive transition. Hence, the emission originates from the anti-Kasha D_2_ → D_0_ transition (Figure 1a) with a much smaller wavelength in the visible region. The second mechanism of emission is based on a hypothesis of hydrogen addition. Within such a mechanism, radicals are formed by adding a hydrogen atom, donated by hydroxyl groups from the PVA matrix to the exited n-substituted benzene dopant in a triplet state.^26^ The resulting visible light emission comes from a D_1_ → D_0_ radiative transition in neutral doublet radicals (Figure 1b). The experimentally recorded differences between the radical emission lifetimes complicate the situation even further. For seemingly similar compounds, in the study of Li et al.,^28^ radical emissions were reported at an order of ≈5.5 ns, while in Yang et al.,^26^ radical emissions were reported at an order of ≈45 ms.

In this study, we investigate both potential radical emission mechanisms with time-dependent density functional theory (TD-DFT) and complete-active space perturbation theory (CASPT2). We investigate absorption and emission properties in 11 different *n*-substituted benzenes, experimentally investigated by Li et al.^28^ and Yang et al.^26^ The results of computational studies are carefully analyzed and compared to existing experiments.

## Methods

Electronic structures of various compounds were calculated with density functional theory implemented in Gaussian 16 software.^32^ All simulations were conducted with range-separated functional ωB97XD^33^ with a 6-31G(d) basis set. We used the Tamm-Dankoff approximation^34^ to simulate adsorption properties and exited states. For accuracy check, we used LC-ωhPBE^35^ functional to calculate emission on D_2_ excited state geometries previously obtained with ωB97XD. All structures were fully optimized followed by the frequency checks to verify the stability of calculated minimums. For energy calculation of data in the energy diagram, we used global hybrid B3LYP^36,37^ instead of range-separated functionals, as was suggested in previous studies^38,39^.

The molecular structure optimization of the singlet and doublet ground and excited states (S_0_, S_1_, S_2_, D_0_, D_1_, D_2_) of carbonyl-substituted benzene radicals in the different degrees of oxidation (anionic, cationic, and neutral forms) were performed at the complete-active space perturbation theory^40^ (CASPT2) level and TZVPP^41^ basis set using Bagel software.^42^ The calculations needed for the CASPT2 geometry optimizations and energy and oscillator strength evaluations had six active electrons in six active orbitals. Nonadiabatic coupling elements (NACME) between the studied ground and excited states at the equilibrium D_2_ or S_2_ geometries were calculated using the CASPT2 method. The CASPT2 method was used in the calculations of the radiative rate constants *k*_R_ and the internal conversion rate constants *k*_IC_ for the D_2_ → D_0_, D_1_ → D_0_, and D_2_ → D_1_ transitions. Overall, the *k*_R_ and *k*_IC_ rate constants were calculated using the method described in the following refs (43−46).

## Results and Discussion

The emission mechanism is always a consequence of a molecule’s electronic structure, which originates from its chemical structure, geometry, and molecular surroundings. To clarify the difference between the anion and hydrogenated radical hypothesis (Figure 1), as an object of interest, we chose to study the Ph-3COOH compound, reported in Yang et al.^26^ as a prominent example of a light-emitting radical molecule. We analyze the same molecule Ph-3COOH in the form of an anion (Figure 1a) and a hydrogenate neutral radical (Figure 1b). Spin-density plots for both radical species exhibit delocalization of unpaired electrons across the whole molecules, generally indicating their stability (especially in the presence of an H-bond matrix, which provides even more delocalization through noncovalent interactions).^31^

The geometry changes cause electronic structure variations, which can be observed in the molecular orbital diagrams (Figure 1). Single-occupied molecular orbital SOMO(α55) in an anion doublet Ph-3COOH molecule is located close to the lowest unoccupied molecular orbital LUMO(α56) (energy difference 1.1 eV) and far away from the highest double-occupied molecular orbital HdOMO(α54) with an energy difference of 4.27 eV with the next occupied energy level with the same spin. In the hydrogenated neutral Ph-3COOH molecule (Figure 1b), the SOMO(α55)-LUMO(β55) energy difference is larger and equals 2.04 eV with an even larger 3.73 eV gap between same-spin SOMO(α55)-LUMO(α56). This observation reflects simulated emission energies of the D_1_ → D_0_ transition, which are negligibly small for anion radical species and sufficiently large for neutral radicals. Such differences in the electronic structure result in qualitatively different emission mechanisms.

In the case of anion radicals (Figure 1a), as a consequence of the smaller SOMO(α55)-LUMO(α56) gap, the transition energy between D_1_ (predominantly of α55-α56) configuration) and D_0_ states is ≈0.23 eV, i.e., not just far from a visible region but also has a small oscillator strength of *f* = 0.001. The main contribution to emission in the visible range comes, in fact, from the transition between D_2_ (predominantly α55-α57 configuration) and D_0_ states with energy 1.78 eV (λ = 699 nm) with a noticeably strong oscillator strength *f* = 0.12. Such emission corresponds to anti-Kasha emission.^28^ In the case of the hydrogenated radical (Figure 1b), because of the larger SOMO(α55)-LUMO(α56) gap, the energy of the D_1_ → D_0_ transition is 2.57 eV which is equivalent to λ = 482 nm. In this case, no anti-Kasha behavior is observed, and emission could be explained straightforwardly as a D_1_ → D_0_ transition that occurs in the neutral radical after attachment of a hydrogen atom to the Ph-3COOH molecule while the oscillator strength was predicted to be very small *f* ≈ 0.0005.

Despite the planned studies of two radical emission mechanisms, we need to analyze other potential sources of emission. The formation of radicals through Hydrogen addition can occur in multiple ways (Figure S1), starting from electron transfer, proton transfer, or concerted electron–proton transfer. Even though protonation of the benzene ring is not a probable event, we calculated possible compounds formed during the reaction and compared them in Figure S1a for Ph-3COOH as an example. Emission of the initial neutral compound Ph-3COOH occurs at a wavelength of 259 nm and a negligibly small oscillator strength, which undermines its emissive capabilities. If hydrogenation occurs starting with proton transfer, then an intermediate compound in the form of hydrogenated cation Ph-3COOH is formed. Its ground state is singlet, which potentially results in S_1_ → S_0_ emission at λ = 560 nm and almost <10^–5^ oscillator strength. Thus, the hydrogenated cation cannot be responsible for the experimentally recorded 45 ms emission. In the case of a reaction starting from electron transfer, the anion radical is the intermediate compound, which demonstrates D_2_ → D_0_ emission and was discussed above. Other potential candidates are different isomers of Ph-3COOH or products of excited state intramolecular proton transfer. Because of the simplicity of the chemical structure of studied compounds, the stability of their potential isomers is questionable. Most of the isomers we tried to simulate resulted in unsuccessful convergence. Despite this, to completely disregard other potential emission sources except for anion or hydrogenated radicals, we calculated several isomers and rotamers, depicted in Figure S1b. All of them showed emission at wavelengths smaller than the experimental value λ_exp_ = 571 nm with a small oscillator strength. Some of the isomers exhibit anti-Kasha S_3_ → S_0_ or S_2_ → S_0_ emission (Figure S1b). Interestingly, isomer number 1 specifically demonstrated S_1_ → S_0_ emission at λ = 578 nm and oscillator strength 0.0035, which is close to the experimental data. However, we should note that the geometry of this isomer is less stable than the initial Ph-3COOH, with the hydrogen atom hopping from the −CO group on the other side of the molecule forming a bond with the carbon atom belonging to the benzene ring. There is no evidence of a large number of such isomers formed as well as cyclability of the UV-stimulated emission process. Thus, only anion and neutral radical emission mechanisms remain to be discussed.

**Figure 2:**
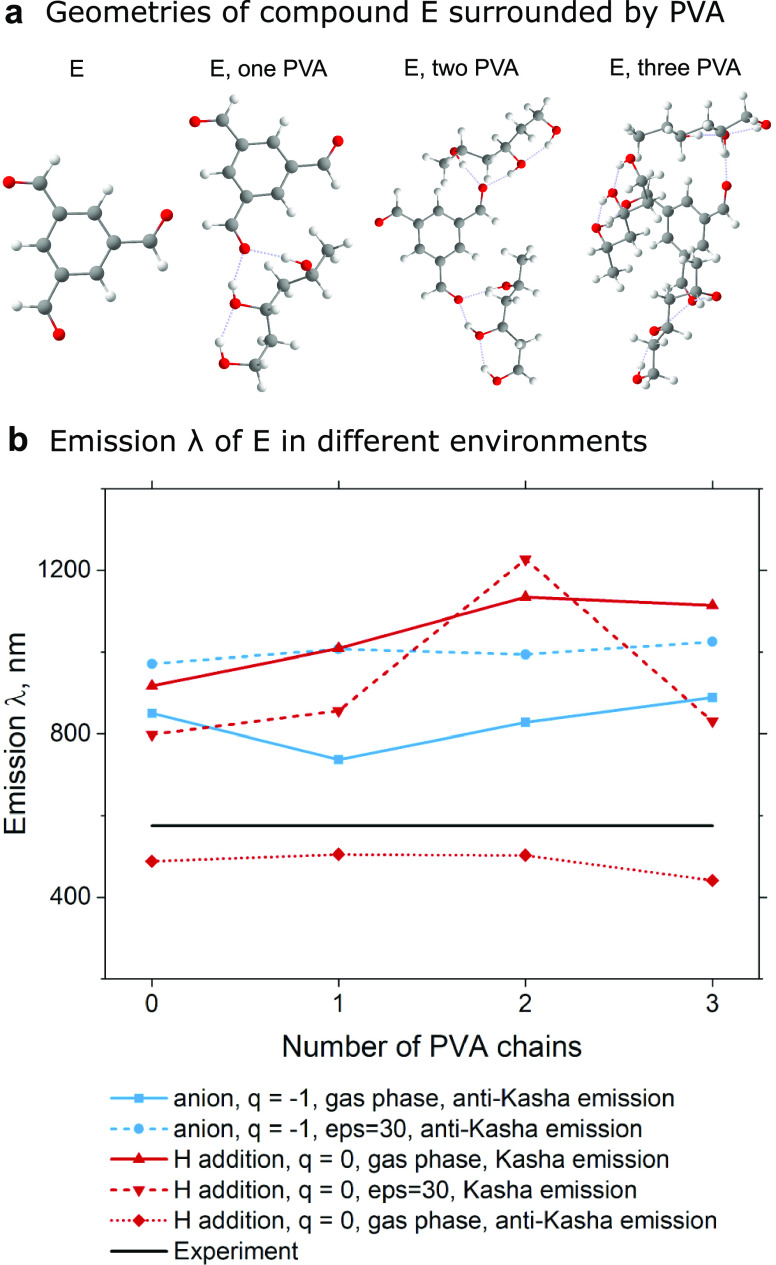
Simulation of compound E under various conditions. We calculated emission in compound E according to an anion and hydrogenated mechanism in the gas phase and PCM model with epsilon 30, depending on the number of PVA chains around. The geometry of anion E with PVA chains is depicted in (a), while emission wavelength data is in (b).

At the next stage, we applied both proposed emission mechanisms to compound E and investigated how the simulated emission wavelength depends on the presence of implicit polarization and explicit PVA matrix chains. The geometry of compound E with a different number of PVA chains can be seen in Figure 2. Each PVA chain forms a hydrogen bond with the carbonyl group in compound E (Figure 2a). The experimentally recorded emission wavelength of compound E in the PVA matrix was measured as λ_exp_ = 575 nm.^28^ Both mechanisms overestimate wavelength compared to the experimentally recorded one (solid blue and red lines in Figure 2b and data in Tables S3 and S4). An addition of PVA chains into simulations corrects the emission wavelengths by taking into account molecular surroundings. Specifically, in the anion emission mechanism, compound E with one PVA chain comes close to the experimental value with λ = 737 nm, which still overestimates experimental data on ≈140 nm. On the other hand, simulations of the D_1_ → D_0_ transition wavelength in hydrogenated neutral compound E, regardless of the PVA surrounding, overestimate the experiment in more than 400 nm, which is highly unrealistic (Table S4). Also, adding polarization into account with ε = 30 (dashed line in Figure 2b) did not lead to quantitive enhancement of the results (Tables S5 and S6).

To our surprise, an unexpectedly good agreement in emission wavelengths appears if we assume the anti-Kasha nature of neutral hydrogenated radicals. For example, hydrogenated E with one PVA chain exhibits D_2_ → D_0_ emission at λ = 504 nm, which is very close to the experimentally reported value of λ_exp_ = 575 nm. Even though such behavior is not typical in simulated compounds, some of them might show anti-Kasha emission even under neutral hydrogenated conditions.

**Figure 3:**
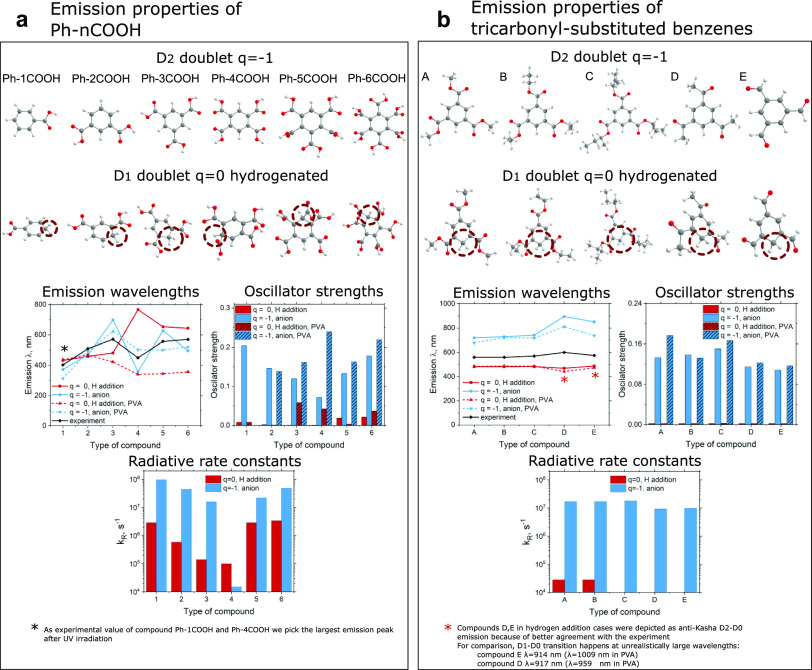
Geometries, emission wavelengths, oscillator strengths, and radiative rate constants of anion and protonated version of (a) Ph-nCOOH and (b) A, B, C, D, E compounds.

Now we apply both the above-mentioned mechanisms of radical emission to 11 different compounds in total, reported by Li et al.^28^ and Yang et al.^26^ For maintaining inheritance, we will keep the same compound naming as in the original papers and split them into two subgroups. The compounds from ref (26) are named Ph-nCOOOH, where *n* = 1, 2, 3, 4, 5, 6 (Figure 3a), and the compounds from ref (28) are named A, B, C, D, E (Figure 3b). A detailed summary of emission properties can be found in supporting Tables S7–S12 (Tables S7a and 8a contain data, simulated with the LC-ωhPBE functional).

All compounds were simulated in the gas phase both alone and in the presence of one PVA chain. In the case of Ph-nCOOH, we can conclude that generally, the anion radical anti-Kasha emission mechanism agrees with the experiment better than the neutral hydrogenated mechanism. The presence of the PVA chain makes an essential correction of emission wavelengths and shows that taking into account the molecular environment is an important step in obtaining the correct results (Figure 3a). In the case of compounds A, B, C, D, and E, the situation is a bit different. Hydrogenated radicals better agree with the experimental data and exhibit a slight underestimation of emission wavelengths. In contrast, the anion radical model overestimates more than 100 nm experimental results. While the presence of the PVA chain corrects anion radical emission to some extent, the hydrogenated neutral radical model is still “energetically” better (Figure 3b). However, hydrogenated neutral compounds E and D agree with the experiment only under the condition that we assume D_2_ → D_0_ anti-Kasha emission (marked by an asterisk in Figure 3b). The D_1_ → D_0_ emission wavelength is between 900 and 1000 nm, which exceeds the experimental results (see Supporting Information, Table S9).

Oscillator strength is directly connected with the luminescence radiative lifetime. Simulations made for anion radicals show large and noticeable oscillator strengths regardless of the presence of PVA chains. On the other hand, in the case of neutral hydrogenated radicals, the calculated oscillator strengths were negligibly low (<0.0001). Only after adding the PVA chain to the simulation did some hydrogenated radicals begin to have noticeable oscillator strengths (≈0.01–0.05, Figure 3a). However, most of them (for instance, all trisubstituted benzenes A, B, C, D, and E) even in the presence of PVA remained “dark”.

The radiative rate constant can be extracted according to the Stricker–Berg equation^47,48^
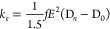
where *f* is the oscillator strength and *E* (cm^–1^) is the energy of D_*n*_ → D_0_ emissive transition (with *n* = 1,2 depending on the emission mechanism). We plot radiative rate constants on a logarithmic scale for all of the compounds at the bottom of Figure 3. For Ph-nCOOH compounds simulated within the anion emission mechanism calculated lifetimes are in order of tens of nanoseconds which contradicts experimentally reported experimental data of tens of milliseconds, reported by Yang et al.^26^ As for hydrogenated compounds, we observe orders of magnitude smaller *k*_r_ compared to anionic (Figure 3a), which still corresponds of tens of microseconds (orders of magnitude less than in experiment, Figure 3a). Similar conclusions can be obtained from the oscillator strengths and radiative rate constant data for D_2_ → D_0_ emission of A–E compounds. Our simulations indicate D_2_ state radiative lifetime as tens of nanoseconds in agreement with the experiment reported by Li et al.,^28^ while the values for hydrogenated counterparts fail to reproduce the experiment (Figure 3b). Oscillator strengths are <10^–4^ for compounds C, D, and E, suggesting negligible radiative rates. More detailed data regarding simulated radiative and nonradiative rate constants, as well as quantum yield values, can be found in supplementary Tables S13–S17.

**Figure 4:**
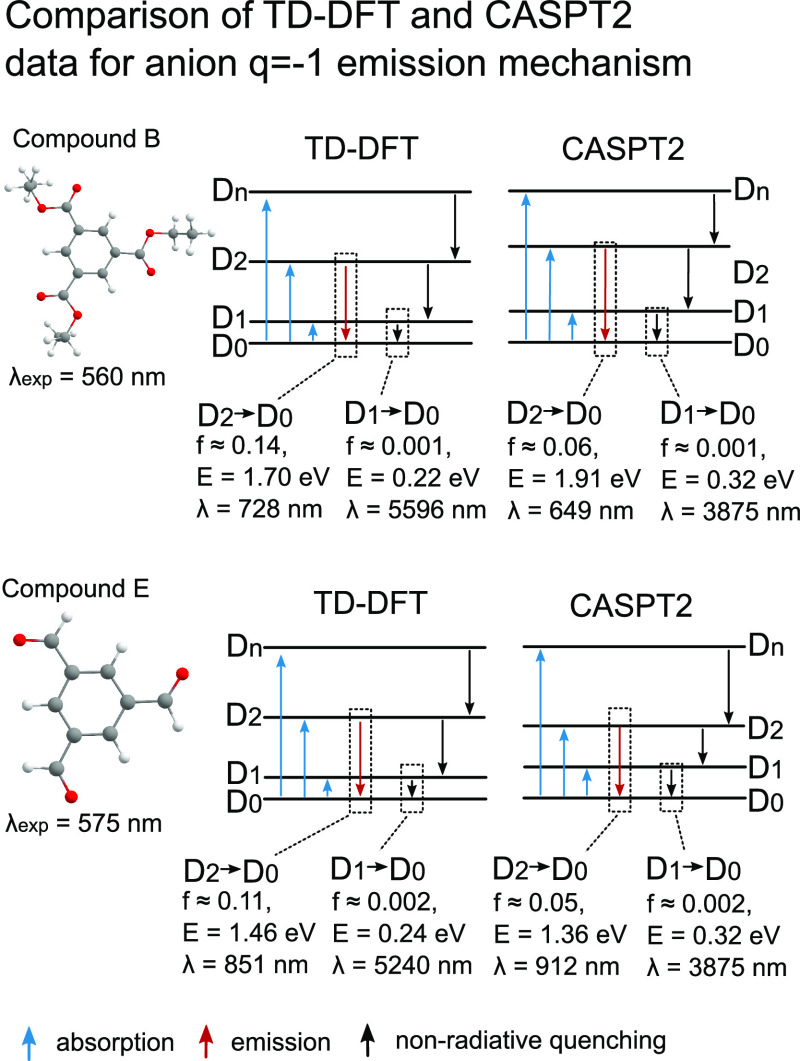
Comparison of emission data calculated with DFT and CASPT2 for compounds B and E.

Unrestricted density functional theory simulations have several issues, which can question the calculated results — mainly the issues with spin contamination and convergence to global minimums during geometrical optimization. We performed more accurate ab initio CASPT2 simulations to verify the DFT results. Because of high computational demand, only two compounds, namely, B and E were simulated within the anion radical emission mechanism (Figure 4). While there was no possibility to investigate the impact of PVA chains on the results, simulations of the emission properties with CASPT2 provide a very good qualitative agreement for the data, obtained with TD-DFT for isolated B and E molecules. For both compounds, the anti-Kasha emission behavior remains intact with a small energy of “dark” D_1_ → D_0_ transition of 0.3 eV and a large energy “bright” D_2_ → D_0_ transition. In the case of compound E, D_2_ → D_0_ emission wavelength is 60 nm larger in the CASPT2 approach, but in the case of compound B, it is 80 nm smaller than TD-DFT data, hence, in better agreement with the experiment. The oscillator strengths for D_1_ → D_0_ and D_2_ → D_0_ transitions in CASPT2 simulations are similar to those in TD-DFT. An essential observation made from CASPT2 calculations relates to the high energies of quartet states, which prevents intersystem crossing between D_2_ and corresponding quartet states (Tables S13 and S14). Overall, we can conclude that TD-DFT simulations are valid and in agreement with the CASPT2 data.

To clearly understand the obtained data, we summarize our thoughts regarding both emission mechanisms in Table 1. While the emission data suggested that the anion mechanism has more solid points to be considered the most important, we do not have any reasons to dismiss the hydrogenated radical mechanism. Both emission mechanisms can coexist at the same time. In case there are sources of hydrogen atoms in a thin film, there could be hydrogenated emissive radicals. Even more, some hydrogenated radicals demonstrate anti-Kasha emission in excellent agreement with the experimental data. This means that anti-Kasha emission is rather a feature of radical emission. However, we argue that the main source of nanosecond emission for compounds A–E originates from the anion radicals and we cannot confirm that millisecond afterglow for compounds Ph-nCOOH originates from their hydrogenated neutral radicals.

**Table 1:** Summary of Arguments for Each of the Emission Mechanisms

arguments for anion *q* = −1 radicals	arguments for H addition *q* = 0 radicals
• resonable oscillator strength and radiative rate constants for all of the compounds. the oscillator strengths of hydrogenated compounds are mostly too small to relate to the experimental data in Li et al.^28^ and if we consider that afterglow at millisecond times, reported in Yang et al.^26^ is of fluorescent origin.	• neutral radicals often demonstrate better agreement in emission wavelength with the experimental data. however, this is valid only if we consider that in some cases hydrogenated compounds demonstrate anti-Kasha emission behavior.
• the anion emission mechanism is valid without chemical modifications, while hydrogenated emission requires the attachment of one individual free hydrogen atom to the aromatic ring. there are cases of radical emission by similar compounds in hydrogen-less environments, such as ionic liquids or PMMA matrices where there is no way of adding Hydrogen atoms to the molecules.^14,31^	• it was reported that the compound Ph-3COOH is not luminescent in poly(methyl methacrylate) (PMMA) and polystyrene (PS) but exhibits luminescent capabilities in polyvinylpyrrolidone (PVP), where active hydrogen atoms are obtained from pyrrolidone’s β-position of carbonyl groups.^26^
• emission activation is repeatable.^29^ under heating, compounds can be reversed into a nonemissive state and repeatedly reactivated again. while it fits with the anion mechanism, repeated detachment and reattachment of Hydrogen atoms require substantial energy because of its covalent bonding nature. It is highly questionable, that “radicals absorb heat from the surroundings^26^” is an explanation of C–H bond rupture. we assume a high probability of appending a second H to the radical under heating, rather than breaking the C–H bond.	• additional experiments to those, reported by Yang et al.^26^ potentially can record fluorescence around 570 nm of 100 ns–10 μs lifetimes in Ph-3,5,6COOH, which can demonstrate excellent agreement with simulations.

## Conclusions

The nature of luminescence in various emissive radicals was discussed within two distinctive mechanisms: emissive anion radicals and emissive neutral hydrogenated radicals. We simulate and analyze 11 different compounds according to both mechanisms to understand which of the mechanisms is correct. Simulated emission wavelengths were compared with the available experimental data.

Calculated results give us a very important perspective on radical emission. Namely, radical emission originates from anion compounds mostly, even though there could be emissive hydrogenated radicals so long as there is a source of Hydrogen atoms in the molecular surrounding. The main emission occurred during the D_2_ → D_0_ radiation transitions. It is important to mention, that anti-Kasha emission can occur not exclusively in anion radicals, but also in neutral hydrogenated radicals.

While data calculated in hydrogenated compounds are promising in wavelength agreement with the experiments, agreement in oscillator strengths and radiative rate constants is questionable and requires additional experiments to investigate the presence of fluorescence of ns—μs lifetimes. This issue can not be corrected by the presence of implicit solvation or molecular surroundings in the form of explicit PVA chains. The anion emission mechanism provides more consistent values of oscillator strengths and radiative rate constants and hence better agreement with the experiment compounds. DFT simulations are in good agreement with CASPT2.

## Supporting Information Available

Analysis of other emission mechanisms on the example of Ph-3COOH; absorption and emission properties calculated using TDA/ωB97XD/6-31G(d) for cation radicals; in neutral compounds, for anion doublet **E** in the PVA matrix, for hydrogenated neutral doublet **E** in the PVA matrix, for anion doublet **E** in the PVA matrix and ε = 30, for hydrogenated neutral doublet **E** in the PVA matrix and ε = 30, for anion radical compounds (LC-ωHPBE), for Ph-nCOOH anion radical compounds (LC-ωHPBE), for hydrogenated neutral doublet compounds, for anion doublet compounds with one PVA chain, for hydrogenated doublet compounds with one PVA chain; CASPT2 simulations of compound **E** and of compound **B**; radiative and nonradiative constants; conversion of experimentally recorded emission wavelengths to energy; radiative and nonradiative rate constants simulated on experimentally measured emission wavelengths (transition energies) (PDF)
