# Supplementary material for: Neutral vs Charged Luminescent Radicals: Anti-Kasha Emission and the Impact of Molecular Surrounding
Source: J Phys Chem A. 2024 Jun 20;128(26):5138–45. doi: 10.1021/acs.jpca.4c02779 (PMC11229066; doi:10.1021/acs.jpca.4c02779)
Supplement: Supplementary file 1 — jp4c02779_si_001.pdf [file jp4c02779_si_001.pdf]

Neutral vs Charged Luminescent Radicals: anti-Kasha Emission and the Impact of Molecular Surrounding

I. Sahalianov[a,b]*, R. R. Valiev[c], R. R. Ramazanov[c], G. Baryshnikov[a,b]*

[a]Laboratory of Organic Electronics, Department of Science and Technology, Linköping University, SE-60174, Norrköping, Sweden

[b]Wallenberg Initiative Materials Science for Sustainability, ITN, Linköping University, 60174 Norrköping,

[c]University of Helsinki, Department of Chemistry, P.O. Box 55 (A.I. Virtanens plats 1), FIN-00014 University of Helsinki, Finland

Corresponding author: ihor.sahalianov@liu.se, glib.baryshnikov@liu.se

SUPPORTING INFORMATION

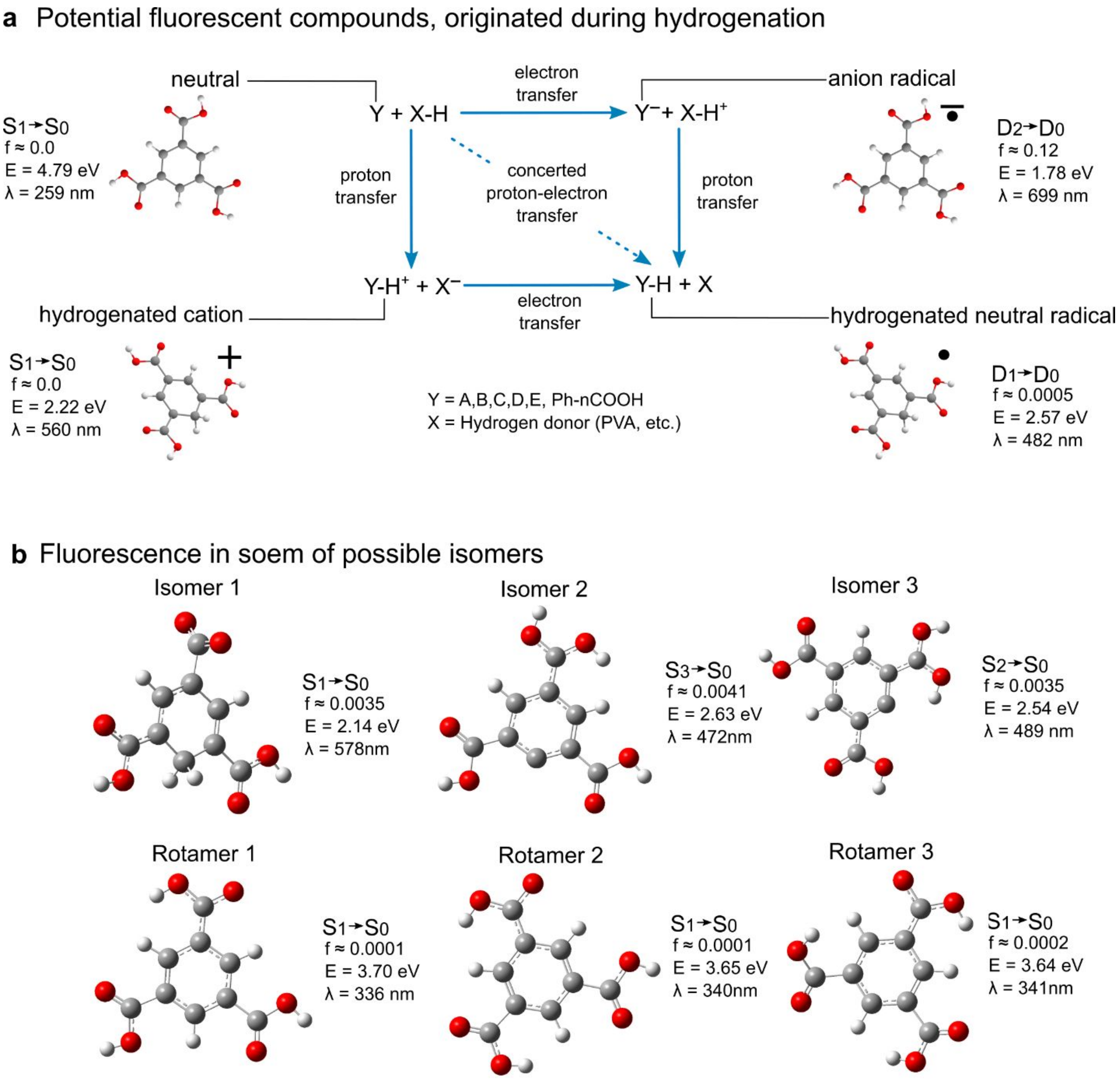

Figure S1 Analysis of other emission mechanisms on the example of Ph-3COOH. **a** — Emission in possible products of hydrogenation through different ways. **b** — Emission of some of the possible isomers and rotamers

Table S1

Absorption and emission properties in cation radical compounds using TDA/ωB97XD/6-31G(d).

Species	Transition	λ, nm	f	Assignment	$\langle S^2 \rangle$
**A**<br>q = +1<br>doublet<br>Optimized geometries of D_0 and D_1 states	D_0-D_1	1480.82	0.0007	53B -> 66B -0.12854<br>60B -> 66B 0.87210<br>62B -> 66B 0.41114	0.806
	D_1-D_0	-2778.24	-0.0001	59B -> 66B 0.33268<br>61B -> 66B 0.27718<br>65B -> 66B -0.86475	0.785

	D_2-D_0	2104.75	0.0001	61B -> 66B -0.19219<br>62B -> 66B 0.59549<br>64B -> 66B 0.72976	0.820
**B**<br>q = +1<br>doublet<br>Optimized geometries of D_0 and D_1 states	D_0-D_1	1430.30	0.0005	70B -> 78B 0.15872<br>72B -> 78B 0.89965<br>74B -> 78B 0.28934	0.807
	D_1-D_0	-2828.58	-0.0003	71B -> 78B 0.37675<br>72B -> 78B -0.20345<br>77B -> 78B 0.85590	0.783
	D_2-D_0	2095.27	0.0003	71B -> 78B 0.15575<br>73B -> 78B -0.22306<br>76B -> 78B -0.90968	0.816
**C**<br>q = +1<br>doublet<br>Optimized geometries of D_0 and D_1 states	D_0-D_1	1406.02	0.0006	91B ->102B -0.25039<br>94B ->102B 0.70723<br>96B ->102B -0.49865	0.807
	D_1-D_0	-2349.76	-0.0003	92B ->102B -0.22935<br>95B ->102B 0.26660<br>101B ->102B 0.86256	0.783
	D_2-D_0	2413.14	0.0021	96B ->102B 0.27722<br>99B ->102B 0.73933<br>100B ->102B 0.39029	0.820
**D**<br>q = +1<br>doublet<br>Optimized geometries of D_0 and D_1 states	D_0-D_1	1504.30	0.0043	51B -> 54B 0.15439<br>52B -> 54B 0.21611<br>53B -> 54B 0.94348	0.832
	D_1-D_0	-3133.28	0.0000	51B -> 54B -0.52676<br>52B -> 54B -0.78968<br>53B -> 54B -0.18661	0.864
	D_2-D_0	3921.82	0.0090	51B -> 54B -0.26278<br>51B -> 55B -0.10566<br>52B -> 54B 0.81768<br>53B -> 54B 0.43494	0.833
**E**<br>q = +1<br>doublet<br>Optimized geometries of D_0 and D_2 states	D_0-D_1	2333.39	0.0000	32B -> 42B -0.22491<br>37B -> 42B 0.34790<br>38B -> 42B 0.88777	0.765
	D_1-D_0	1827.93	0.0084	38B -> 42B -0.71898<br>39B -> 42B 0.53532<br>41B -> 42B -0.40707	0.781
	D_2-D_0				

Table S2

Absorption and emission properties in neutral compounds using TDA/ωB97XD/6-31G(d).

Species	Transition	λ, nm	f	Assignment	<S^2>
**A**<br>q = 0<br>singlet	S_0-S_1	246.83	0.0000	65 -> 67 0.36919<br>66 -> 67 -0.32728<br>66 -> 68 0.36917	0.0
	S_1-S_0	259.06	0.0000	65 -> 67 -0.48551<br>66 -> 68 -0.48551	0.0
**B**<br>q = 0<br>singlet	S_0-S_1	246.93	0.0000	77 -> 80 0.46794<br>78 -> 79 0.46797<br>78 -> 80 -0.15458	0.0
	S_1-S_0	259.15	0.0000	77 -> 79 -0.25481<br>77 -> 80 0.42246<br>78 -> 79 0.42246	0.0
**C**<br>q = 0<br>singlet	S_0-S_1	246.98	0.0000	101 ->103 0.26438<br>101 ->104 0.41481<br>102 ->103 0.41502	0.0
	S_1-S_0	259.23	0.0000	101 ->103 0.24502<br>101 ->104 0.42709<br>102 ->103 0.42764	0.0
**D**<br>q = 0<br>singlet	S_0-S_1	311.54	0.0001	50 -> 57 -0.32278<br>53 -> 55 -0.35470<br>54 -> 56 0.35509	0.0

	S_1-S_0	374.00	0.0001	54 -> 55 0.62055<br>54 -> 57 0.27066<br>54 -> 59 -0.14968	0.0
**E**<br>q = 0<br>singlet	S_0-S_1	320.42	0.0001	38 -> 45 -0.32558<br>41 -> 43 0.41050<br>42 -> 44 -0.41004	0.0
	S_1-S_0	375.94	0.0001	39 -> 43 0.13753<br>42 -> 43 0.61041<br>42 -> 45 0.27321	0.0

Table S3

Absorption and emission properties in anion doublet compound **E** in PVA matrix using TDA/ωB97XD/6-31G(d).

Species	Transition	λ, nm	f	Assignment	<S^2>
**E**<br>q = -1, doublet<br>One PVA chain<br>Optimized D_0 geometry	D_0-D_1	1244.05	0.0000	80A -> 81A 0.97688<br>80A -> 84A 0.11814	0.817
	D_0-D_2	600.48	0.099	80A -> 82A 0.95219<br>79B -> 81B -0.19425	0.810
**E**<br>q = -1, doublet<br>One PVA chain<br>Optimized D_2 geometry	D_1-D_0	2065.06	0.0008	80A -> 81A 0.97937<br>80A -> 84A 0.11719	0.809
	D_2-D_0	736.67	0.117	80A -> 82A 0.95925<br>77B -> 80B 0.14438<br>79B -> 81B -0.12868	0.809
**E**<br>q = -1, doublet<br>Two PVA chains<br>Optimized D_0 geometry	D_0-D_1	1583.64	0.0172	117A ->118A 0.93255<br>117A ->119A 0.30040<br>117A ->121A 0.10569	0.818
	D_0-D_2	681.88	0.0994	117A ->118A -0.28291<br>117A ->119A 0.90935<br>116B ->118B -0.15867	0.830
**E**<br>q = -1, doublet<br>Two PVA chains<br>Optimized D_2 geometry	D_1-D_0	2584.54	0.012	117A ->118A 0.91651<br>117A ->119A 0.34900<br>117A ->121A 0.11300	0.811
	D_2-D_0	827.96	0.109	117A ->118A -0.32927<br>117A ->119A 0.89693<br>116B ->117B 0.20460	0.832
**E**<br>q = -1, doublet<br>Three PVA chains<br>Optimized D_0 geometry	D_0-D_1	1423.66	0.0067	154A ->155A 0.95968<br>154A ->156A 0.19385<br>154A ->158A -0.10139	0.811
	D_0-D_2	644.07	0.0896	154A ->155A -0.18777<br>154A ->156A 0.93808<br>153B ->155B -0.17770	0.816
**E**<br>q = -1, doublet<br>Three PVA chains<br>Optimized D_2 geometry	D_1-D_0	3371.63	0.006	154A ->155A -0.94778<br>154A ->156A -0.25428	0.801
	D_2-D_0	889.07	0.0983	154A ->155A 0.24040<br>154A ->156A -0.92801<br>153B ->154B 0.18801	0.834

Table S4

Absorption and emission properties in neutral hydrogenated doublet compound **E** in PVA matrix using TDA/ωB97XD/6-31G(d).

Species	Transition	λ, nm	f	Assignment	<S^2>
**E**<br>q = 0, doublet	D_0-D_1	508.24	0.0000	76B -> 80B 0.43088<br>76B -> 81B 0.30448<br>77B -> 80B 0.69178	1.316

One PVA chain Optimized D_0 geometry	D_0-D_2	484.84	0.0000	76B -> 80B 0.69021<br>77B -> 80B -0.41813<br>77B -> 81B 0.26194	1.280
**E**<br>q = 0, doublet<br>One PVA chain Optimized D_1 geometry	D_1-D_0	1008.88	0.0000	77B -> 80B -0.85349<br>77B -> 81B 0.40970<br>77B -> 82B -0.22143	1.024
	D_2-D_0	502.37	0.0157	80A -> 81A 0.51512<br>75B -> 80B 0.50841<br>76B -> 80B -0.60885	1.030
**E**<br>q = 0, doublet<br>One PVA chain Optimized D_2 geometry	D_1-D_0	896.31	0.0000	77B -> 80B -0.84165<br>77B -> 81B 0.43510<br>77B -> 82B -0.21486	0.947
	D_2-D_0	504.83	0.0094	80A -> 81A 0.56902<br>75B -> 80B 0.73512<br>76B -> 80B -0.24214	1.094
**E**<br>q = 0, doublet<br>Two PVA chains Optimized D_0 geometry	D_0-D_1	553.57	0.0000	109B ->117B 0.23038<br>113B ->117B 0.81221<br>113B ->119B 0.31052	1.122
	D_0-D_2	479.53	0.0000	110B ->117B 0.70677<br>110B ->118B -0.30142<br>111B ->117B -0.30453	1.377
**E**<br>q = 0, doublet<br>Two PVA chains Optimized D_1 geometry	D_1-D_0	1135.25	0.0000	115B ->117B 0.85607<br>115B ->119B -0.34402<br>116B ->117B -0.18600	1.004
	D_2-D_0	475.23	0.0000	110B ->117B 0.78930<br>110B ->118B -0.39574<br>110B ->119B 0.34239	1.422
**E**<br>q = 0, doublet<br>Two PVA chains Optimized D_2 geometry	D_1-D_0	902.95	0.0000	113B ->117B -0.82694<br>113B ->118B -0.41072<br>113B ->119B -0.25864	1.084
	D_2-D_0	502.47	0.0021	117A ->118A 0.52175<br>109B ->117B 0.67955<br>111B ->117B 0.24396	1.114
**E**<br>q = 0, doublet<br>Three PVA chains Optimized D_0 geometry	D_0-D_1	509.53	0.0001	145B ->154B -0.31769<br>146B ->154B -0.26000<br>147B ->154B 0.72155	1.128
	D_0-D_2	476.82	0.0003	154A ->155A 0.69385<br>142B ->154B -0.16920<br>144B ->154B -0.61475	0.871
**E**<br>q = 0, doublet<br>Three PVA chains Optimized D_1 geometry	D_1-D_0	1114.13	0.0000	152B ->154B -0.50607<br>153B ->154B -0.73334<br>153B ->156B 0.30989	1.009
	D_2-D_0	452.16	0.0005	154A ->155A 0.25844<br>145B ->154B -0.25989<br>146B ->154B -0.64019	1.295
**E**<br>q = 0, doublet<br>Three PVA chains Optimized D_2 geometry	D_1-D_0	1113.47	0.0000	152B ->154B 0.46195<br>153B ->154B -0.76175<br>153B ->156B 0.32228	1.009
	D_2-D_0	440.97	0.0010	154A ->155A -0.56415<br>142B ->154B 0.58440<br>144B ->154B 0.27487	1.100

Table S5

Absorption and emission properties in anion doublet compound **E** in PVA matrix and implicit solvent with dielectric permittivity ε=30 using TDA/ωB97XD/6-31G(d).

Species	Transition	λ, nm	f	Assignment	<S^2>
**E**<br>q = -1, doublet<br>Optimized D_0 geometry	D_0-D_1	2006.08	0.0022	43A -> 44A 0.98160<br>43A -> 47A 0.13027	0.809
	D_0-D_2	716.74	0.1246	43A -> 45A 0.96099<br>40B -> 43B -0.15388<br>42B -> 44B -0.14444	0.819

**E**<br>q = -1, doublet<br>Optimized D_2 geometry	D_1-D_0	3480.01	0.0041	43A -> 44A 0.98343<br>43A -> 46A 0.12524	0.796
	D_2-D_0	971.80	0.1830	43A -> 45A -0.97436	0.822
**E**<br>q = -1, doublet<br>One PVA chain<br>Optimized D_0 geometry	D_0-D_1	1105.10	0.0004	80A -> 81A 0.97749<br>80A -> 84A -0.12619	0.818
	D_0-D_2	577.49	0.1113	80A -> 82A 0.95560<br>79B -> 81B -0.19270	0.812
**E**<br>q = -1, doublet<br>One PVA chain<br>Optimized D_2 geometry	D_1-D_0	5313.59	0.0035	80A -> 81A 0.98286<br>80A -> 85A 0.11854	0.794
	D_2-D_0	1007.08	0.1487	80A -> 82A 0.97159<br>79B -> 80B 0.13770	0.821
**E**<br>q = -1, doublet<br>Two PVA chains<br>Optimized D_0 geometry	D_0-D_1	1338.19	0.0068	117A ->118A 0.95653<br>117A ->119A 0.21064<br>117A ->121A -0.10718	0.813
	D_0-D_2	637.83	0.1085	117A ->118A -0.20593<br>117A ->119A 0.93773<br>116B ->118B -0.16872	0.811
**E**<br>q = -1, doublet<br>Two PVA chains<br>Optimized D_2 geometry	D_1-D_0	3366.57	0.0150	117A ->118A 0.94917<br>117A ->119A 0.26164<br>117A ->121A 0.10393	0.809
	D_2-D_0	994.17	0.1417	117A ->118A -0.25842<br>117A ->119A 0.93668<br>116B ->117B 0.14466	0.813
**E**<br>q = -1, doublet<br>Three PVA chains<br>Optimized D_0 geometry	D_0-D_1	1017.76	0.0009	154A ->155A 0.96702<br>154A ->156A 0.13147<br>154A ->158A 0.12198	0.824
	D_0-D_2	558.24	0.1004	154A ->155A -0.12593<br>154A ->156A 0.94588<br>153B ->155B -0.16603	0.822
**E**<br>q = -1, doublet<br>Three PVA chains<br>Optimized D_2 geometry	D_1-D_0	4007.39	0.0095	154A ->155A -0.95974<br>154A ->156A -0.21830<br>154A ->158A -0.10463	0.804
	D_2-D_0	1025.59	0.1347	154A ->155A 0.21552<br>154A ->156A -0.94729<br>153B ->154B -0.11154	0.818

Table S6

Absorption and emission properties in neutral hydrogenated doublet compound **E** in PVA matrix and implicit solvent with dielectric permittivity ε=30 using TDA/ωB97XD/6-31G(d).

Species	Transition	λ, nm	f	Assignment	$\langle S^2 \rangle$
**E**<br>q = 0, doublet<br>Optimized D_0 geometry	D_0-D_1	508.94	0.0000	39B -> 45B -0.31857<br>41B -> 43B 0.68251<br>42B -> 43B -0.48850	1.171
	D_0-D_2	479.67	0.0000	39B -> 44B 0.31719<br>41B -> 43B 0.48985<br>42B -> 43B 0.57482	1.338
**E**<br>q = 0, doublet<br>Optimized D_1 geometry	D_1-D_0	798.22	0.0000	40B -> 43B -0.18883<br>42B -> 43B 0.87412<br>42B -> 45B 0.38003	1.010
	D_2-D_0	474.52	0.0000	40B -> 44B 0.40904<br>41B -> 43B 0.77606<br>41B -> 45B -0.30396	1.425
**E**<br>q = 0, doublet<br>One PVA chain<br>Optimized D_0 geometry	D_0-D_1	490.10	0.0000	76B -> 80B 0.61778<br>76B -> 81B 0.32163<br>77B -> 80B -0.53026	1.297
	D_0-D_2	473.95	0.0000	73B -> 80B 0.27301<br>76B -> 80B 0.52377<br>77B -> 80B 0.59173	1.247
**E**<br>q = 0, doublet	D_1-D_0	856.60	0.0000	77B -> 80B -0.84022<br>77B -> 81B 0.40614	1.028

One PVA chain Optimized D_1 geometry				77B -> 82B -0.20035	
	D_2-D_0	506.15	0.0304	80A -> 81A -0.48077<br>75B -> 80B -0.39959<br>76B -> 80B -0.72436	0.988
**E**<br>q = 0, doublet<br>Two PVA chains<br>Optimized D_0 geometry	D_0-D_1	504.81	0.0000	111B ->117B -0.46554<br>112B ->117B -0.40030<br>113B ->117B 0.56014	1.145
	D_0-D_2	477.20	0.0003	117A ->118A 0.70519<br>109B ->117B -0.59699<br>110B ->117B 0.21477	0.861
**E**<br>q = 0, doublet<br>Two PVA chains<br>Optimized D_1 geometry	D_1-D_0	1227.46	0.0001	109B ->117B -0.20223<br>116B ->117B -0.88218<br>116B ->119B 0.32401	0.990
	D_2-D_0	468.38	0.0000	111B ->117B 0.78965<br>111B ->118B -0.38020<br>111B ->119B 0.35112	1.377
**E**<br>q = 0, doublet<br>Three PVA chains<br>Optimized D_0 geometry	D_0-D_1	484.08	0.0004	147B ->154B -0.45751<br>149B ->154B 0.57686<br>152B ->154B -0.34781	1.226
	D_0-D_2	470.83	0.0002	147B ->154B 0.56297<br>149B ->154B 0.49552<br>149B ->155B 0.25375	1.246
**E**<br>q = 0, doublet<br>Three PVA chains<br>Optimized D_1 geometry	D_1-D_0	830.96	0.0000	149B ->154B 0.51589<br>151B ->154B 0.66608<br>151B ->155B -0.28514	1.049
	D_2-D_0	500.13	0.0287	154A ->155A -0.45578<br>145B ->154B 0.60510<br>152B ->154B 0.31309	0.918

Table S7

Absorption and emission properties in anion radical compounds using TDA/ωB97XD/6-31G(d).

Species	Transition	λ, nm	f	Assignment	<S^2>
**A**<br>q = −1<br>doublet<br>Optimized geometries of D_0 and D_2 states	D_0-D_1	2272.65	0.0034	67A -> 68A 0.98315<br>67A -> 73A 0.13141	0.800
	D_0-D_2	627.76	0.1337	67A -> 69A 0.95626<br>65B -> 67B 0.17654<br>66B -> 68B -0.16606	0.835
	D_1-D_0	5580.96	0.0013	67A -> 68A 0.98410<br>67A -> 72A 0.12862	0.784
	D_2-D_0	719.38	0.1328	67A -> 69A 0.95644<br>65B -> 68B 0.11948<br>66B -> 67B 0.21036	0.848
**B**<br>q = −1<br>doublet<br>Optimized geometries of D_0 and D_2 states	D_0-D_1	2260.62	0.0035	79A -> 80A 0.98310<br>79A -> 86A 0.11921	0.800
	D_0-D_2	633.54	0.1394	79A -> 81A 0.95676<br>77B -> 79B 0.17447<br>78B -> 80B -0.16522	0.834
	D_1-D_0	5596.37	0.0014	79A -> 80A -0.98414<br>79A -> 86A -0.11588	0.784
	D_2-D_0	727.83	0.1384	79A -> 81A 0.95705<br>77B -> 80B 0.11821<br>78B -> 79B 0.20761	0.847
**C**<br>q = −1<br>doublet<br>Optimized geometries of D_0 and D_2 states	D_0-D_1	2233.83	0.0038	103A ->104A 0.98278<br>103A ->114A 0.11663	0.799
	D_0-D_2	642.62	0.1504	103A ->105A 0.95715<br>101B ->103B 0.16487<br>102B ->104B -0.15878	0.833
	D_1-D_0	5435.07	0.0017	103A ->104A 0.98383<br>103A ->114A 0.11573	0.784
	D_2-D_0	740.31	0.1505	103A ->105A 0.95787<br>101B ->104B 0.11663<br>102B ->103B 0.20414	0.844

**D**<br>q = −1<br>doublet<br>Optimized geometries of D_0 and D_2 states	D_0-D_1	2423.09	0.0037	55A -> 56A 0.97969<br>55A -> 59A -0.14499	0.806
	D_0-D_2	762.48	0.1151	55A -> 57A 0.95933<br>52B -> 55B 0.16785<br>54B -> 56B -0.12655	0.827
	D_1-D_0	5500.85	0.0017	55A -> 56A 0.97989<br>55A -> 59A -0.14667	0.788
	D_2-D_0	894.73	0.1147	55A -> 57A 0.95918<br>51B -> 56B 0.11175<br>54B -> 55B 0.17652	0.834
**E**<br>q = −1<br>doublet<br>Optimized geometries of D_0 and D_2 states	D_0-D_1	2434.91	0.0034	43A -> 44A 0.98125<br>43A -> 47A 0.13140	0.809
	D_0-D_2	730.52	0.1095	43A -> 45A 0.95366<br>40B -> 43B 0.17869<br>42B -> 44B -0.13455	0.843
	D_1-D_0	5239.65	0.0015	43A -> 44A 0.98184<br>43A -> 47A 0.12709	0.790
	D_2-D_0	850.58	0.1083	43A -> 45A 0.95347<br>42B -> 43B 0.19962<br>42B -> 45B -0.12465	0.850

Table S7a

Emission properties in anion radical compounds using TDA/LC-ωHPBE /6-31G(d) calculated on D_2 excited state geometry, obtained from the ωB97XD optimization.

Species	Transition	λ, nm	f	Assignment	$\langle S^2 \rangle$
**A**<br>q = −1<br>doublet<br>Optimized geometry of D_2 state	D_1-D_0	5650.42	0.0045	67A -> 68A 1.03536<br>67A -> 74A 0.18002<br>67A <- 68A 0.33374	0.796
	D_2-D_0	718.31	0.1361	65A -> 68A 0.12569<br>67A -> 69A 0.95451<br>66B -> 67B 0.24894	0.926
**B**<br>q = −1<br>doublet<br>Optimized geometry of D_2 state	D_1-D_0	5739.09	0.0052	79A -> 80A 1.03821<br>79A -> 86A -0.16288<br>79A <- 80A 0.34165	0.797
	D_2-D_0	729.38	0.1451	77A -> 80A 0.12705<br>79A -> 81A 0.95623<br>78B -> 79B 0.24486	0.922
**C**<br>q = −1<br>doublet<br>Optimized geometry of D_2 state	D_1-D_0	5775.64	0.0066	103A ->104A 1.04180<br>103A ->114A 0.12994<br>103A <-104A 0.35058	0.798
	D_2-D_0	746.19	0.1625	101A ->104A 0.12807<br>103A ->105A 0.95897<br>102B ->103B 0.23928	0.914
**D**<br>q = −1<br>doublet<br>Optimized geometry of D_2 state	D_1-D_0	6813.37	0.0088	55A -> 56A 1.07835<br>55A -> 59A 0.20262<br>55A <- 56A 0.45396	0.810
	D_2-D_0	925.08	0.1109	55A -> 57A 0.96670<br>54B -> 55B 0.21004<br>55A <- 57A 0.14273	0.885
**E**<br>q = −1<br>doublet<br>Optimized geometry of D_2 state	D_1-D_0	5830.89	0.0079	43A -> 44A 1.05473<br>43A <- 44A 0.38842	0.814
	D_2-D_0	887.19	0.1027	43A -> 45A 0.96119<br>42B -> 43B 0.23716<br>43A <- 45A 0.14239	0.907

Table S8

Absorption and emission properties in anion doublet compounds using TDA/ωB97XD/6-31G(d).

Species	Transition	λ, nm	f	Assignment	<S^2>
**Ph-1COOH**<br>q = -1<br>doublet<br>Optimized geometries of D_0 and D_2 states	D_0-D_1	690.30	0.0022	33A -> 34A 0.98771	0.797
	D_0-D_2	346.55	0.0876	33A -> 35A 0.85115<br>31B -> 34B 0.10003<br>32B -> 33B 0.49998	0.786
	D_1-D_0	1552.84	0.0009	33A -> 34A 0.99129	0.843
	D_2-D_0	372.29	0.2044	33A -> 35A 0.93009<br>31B -> 34B 0.20542<br>32B -> 33B 0.24775	0.786
**Ph-2COOH**<br>q = -1<br>doublet<br>Optimized geometries of D_0 and D_2 states	D_0-D_1	1153.55	0.0333	44A -> 45A 0.95290<br>44A -> 46A 0.25392<br>43B -> 45B 0.11271	0.828
	D_0-D_2	403.98	0.1022	44A -> 46A 0.89687<br>42B -> 44B 0.21445<br>43B -> 45B -0.29215	0.895
	D_1-D_0	1588.51	0.0230	44A -> 45A -0.94104<br>44A -> 46A -0.30557	0.807
	D_2-D_0	468.86	0.1464	44A -> 45A 0.26686<br>44A -> 46A -0.90267<br>43B -> 44B 0.24039	0.848
**Ph-3COOH**<br>q = -1<br>doublet<br>Optimized geometries of D_0 and D_2 states	D_0-D_1	2273.67	0.0029	55A -> 56A 0.98418<br>55A -> 62A -0.12516	0.801
	D_0-D_2	613.85	0.1207	55A -> 57A 0.95438<br>53B -> 55B 0.18195<br>54B -> 56B -0.16879	0.843
	D_1-D_0	5502.07	0.0010	55A -> 56A 0.98509<br>55A -> 62A 0.12277	0.784
	D_2-D_0	698.49	0.1197	55A -> 57A 0.95433<br>53B -> 56B 0.12331<br>54B -> 55B 0.22051	0.855
**Ph-4COOH**<br>q = -1<br>doublet<br>Optimized geometries of D_0 and D_2 states	D_0-D_1	689.64	0.0020	66A -> 67A 0.97616<br>66A -> 70A 0.15463	0.790
	D_0-D_2	449.47	0.1853	66A -> 68A 0.92660<br>64B -> 66B 0.25205<br>65B -> 67B -0.23894	0.856
	D_1-D_0	1151.15	0.0003	66A -> 67A 0.98003<br>66A -> 70A 0.16052	0.778
	D_2-D_0	358.49	0.0715	65A -> 67A -0.12495<br>66A -> 69A 0.96440<br>65B -> 66B 0.14863	0.841
**Ph-5COOH**<br>q = -1<br>doublet<br>Optimized geometries of D_0 and D_2 states	D_0-D_1	777.30	0.0020	77A -> 78A 0.97343<br>77A -> 82A 0.13050	0.790
	D_0-D_2	455.41	0.1773	77A -> 79A 0.92242<br>75B -> 77B 0.20664<br>76B -> 77B -0.11864<br>76B -> 78B 0.23279	0.856
	D_1-D_0	3126.62	0.0007	77A -> 78A -0.98508	0.783
	D_2-D_0	627.51	0.1332	77A -> 79A -0.95042<br>75B -> 78B -0.12185<br>76B -> 77B -0.22653	0.864
**Ph-6COOH**<br>q = -1<br>doublet<br>Optimized geometries of D_0 and D_2 states	D_0-D_1	714.81	0.0000	88A -> 89A 0.97702<br>88A -> 94A -0.12781	0.798
	D_0-D_2	446.31	0.1430	88A -> 90A 0.91503<br>86B -> 89B -0.12678<br>87B -> 88B -0.30368	0.825
	D_1-D_0	935.44	0.0000	88A -> 89A 0.98165<br>88A -> 94A -0.11184	0.794
	D_2-D_0	495.54	0.1780	88A -> 90A 0.94097<br>86B -> 89B -0.17449<br>87B -> 88B 0.22412	0.831

Table S8a

Emission properties in anion radical compounds using TDA/LC-ωHPBE /6-31G(d) calculated on D_2 excited state geometry, obtained from the ωB97XD optimization.

Species	Transition	λ, nm	f	Assignment	$\langle S^2 \rangle$
**Ph-1COOH**<br>q = -1<br>doublet<br>Optimized geometries of D_0 and D_2 states	D_1-D_0	1468.28	0.0005	33A -> 34A 0.99766<br>33A <- 34A 0.12175	0.801
	D_2-D_0	394.60	0.2089	32A -> 34A -0.24511<br>33A -> 35A 0.88782<br>31B -> 33B -0.36190	1.154
**Ph-2COOH**<br>q = -1<br>doublet<br>Optimized geometries of D_0 and D_2 states	D_1-D_0	1742.85	0.0288	44A -> 45A 0.96158<br>44A -> 46A 0.28673<br>44A <- 45A 0.15099	0.858
	D_2-D_0	471.89	0.1301	44A -> 45A -0.25076<br>44A -> 46A 0.90385<br>43B -> 44B -0.26635	0.910
**Ph-3COOH**<br>q = -1<br>doublet<br>Optimized geometries of D_0 and D_2 states	D_1-D_0	5407.32	0.0033	55A -> 56A 1.03095<br>55A -> 62A 0.17469<br>55A <- 56A 0.31582	0.795
	D_2-D_0	697.68	0.1177	55A -> 57A 0.95158<br>54B -> 55B 0.26046<br>55A <- 57A 0.11176	0.940
**Ph-4COOH**<br>q = -1<br>doublet<br>Optimized geometries of D_0 and D_2 states	D_1-D_0	1150.46	0.0002	66A -> 67A 0.97917<br>66A -> 70A 0.17981	0.788
	D_2-D_0	491.25	0.2148	66A -> 68A 0.94371<br>64B -> 66B 0.15428<br>65B -> 67B 0.24703	0.896
**Ph-5COOH**<br>q = -1<br>doublet<br>Optimized geometries of D_0 and D_2 states	D_1-D_0	2948.70	0.0020	77A -> 78A 0.99750<br>77A <- 78A 0.18366	0.792
	D_2-D_0	623.50	0.1307	76A -> 78A 0.11273<br>77A -> 79A 0.94200<br>76B -> 77B 0.27256	0.964
**Ph-6COOH**<br>q = -1<br>doublet<br>Optimized geometries of D_0 and D_2 states	D_1-D_0	938.05	0.0000	88A -> 89A 0.97325<br>88A -> 94A -0.14366	0.834
	D_2-D_0	504.58	0.2114	87A -> 89A 0.18961<br>88A -> 90A 0.89542<br>86B -> 89B -0.30635	1.101

Table S9

Absorption and emission properties hydrogenated neutral doublet compounds using TDA/ωB97XD/6-31G(d).

Species	Transition	λ, nm	f	Assignment	$\langle S^2 \rangle$
**A**<br>q = 0<br>doublet	D_0-D_1	446.58	0.0002	67A -> 68A 0.69625<br>67A -> 71A -0.10502<br>66B -> 67B 0.68249	0.835
	D_1-D_0	482.11	0.0001	67A -> 68A 0.71430<br>66B -> 67B 0.66977	0.812
**B**<br>q = 0<br>doublet	D_0-D_1	446.81	0.0002	79A -> 80A 0.69539<br>79A -> 83A -0.10493<br>78B -> 79B 0.68239	0.834
	D_1-D_0	482.41	0.0001	79A -> 80A 0.71319<br>78B -> 79B 0.67034	0.812
**C**<br>q = 0<br>doublet	D_0-D_1	446.97	0.0002	103A ->104A 0.69680<br>103A ->107A -0.10459<br>102B ->103B 0.67899	0.834
	D_1-D_0	482.74	0.0000	103A ->104A 0.71569<br>102B ->103B 0.66640	0.812

**D**<br>q = 0<br>doublet	D_0-D_1	501.46	0.0000	52B -> 57B -0.30922<br>53B -> 55B -0.58918<br>54B -> 55B 0.61570	1.162
	D_1-D_0<br>Optimized geometries of D_1 state	913.93	0.0000	54B -> 55B -0.89316<br>54B -> 57B -0.39069<br>54B -> 58B 0.13858	1.010
	D_2-D_0<br>Optimized geometries of D_2 state	926.48	0.0000	54B -> 55B -0.86290<br>54B -> 56B 0.40635<br>54B -> 57B -0.20325	1.030
	D_1-D_0<br>Optimized geometries of D_2 state	469.56	0.0000	51B -> 55B 0.56902<br>51B -> 57B -0.39482<br>53B -> 55B -0.62838	1.382
**E**<br>q = 0<br>doublet	D_0-D_1	532.46	0.0000	40B -> 45B -0.30797<br>41B -> 43B 0.54368<br>42B -> 43B 0.64688	1.167
	D_1-D_0<br>Optimized geometries of D_1 state	916.86	0.0000	42B -> 43B 0.88541<br>42B -> 45B -0.39233<br>42B -> 46B 0.14176	1.010
	D_2-D_0<br>Optimized geometries of D_2 state	933.05	0.0000	42B -> 43B -0.85047<br>42B -> 44B 0.41736<br>42B -> 45B -0.22261	1.044
	D_1-D_0<br>Optimized geometries of D_2 state	488.00	0.0000	39B -> 43B -0.58728<br>39B -> 45B 0.39995<br>41B -> 43B -0.60416	1.397

Table S10

Absorption and emission properties in hydrogenated neutral doublet compounds using TDA/ωB97XD/6-31G(d).

Species	Transition	λ, nm	f	Assignment	<S^2>
**Ph-1COOH**<br>q = 0<br>doublet	D_0-D_1	394.21	0.0087	33A -> 34A -0.59122<br>32B -> 33B 0.77583<br>32B -> 35B -0.12123	0.841
	D_1-D_0	432.19	0.0082	33A -> 34A -0.59568<br>32B -> 33B 0.77651<br>32B -> 35B -0.11808	0.816
**Ph-2COOH**<br>q = 0<br>doublet	D_0-D_1	423.35	0.0023	44A -> 45A -0.64955<br>44A -> 46A 0.14431<br>43B -> 44B 0.71299	0.836
	D_1-D_0	458.53	0.0019	44A -> 45A -0.65919<br>44A -> 46A 0.12333<br>43B -> 44B -0.71397	0.811
**Ph-3COOH**<br>q = 0<br>doublet	D_0-D_1	446.62	0.0006	55A -> 56A 0.68646<br>55A -> 59A -0.10014<br>54B -> 55B 0.69247	0.836
	D_1-D_0	481.61	0.0005	55A -> 56A 0.69748<br>54B -> 55B 0.68713	0.814
**Ph-4COOH**<br>q = 0<br>doublet	D_0-D_1	501.25	0.0000	65A -> 67A 0.58279<br>66A -> 67A -0.31837<br>65B -> 66B 0.66127	2.725
	D_1-D_0	767.27	0.0009	64B -> 66B 0.41377<br>65B -> 66B 0.80663<br>65B -> 67B -0.26362	0.964
**Ph-5COOH**<br>q = 0<br>doublet	D_0-D_1	515.45	0.0141	77A -> 78A 0.91853<br>72B -> 77B 0.14817<br>76B -> 77B 0.28316	0.826
	D_1-D_0	653.35	0.0190	77A -> 78A -0.95114<br>72B -> 77B 0.10021	0.805

				76B -> 77B 0.22344	
**Ph-6COOH**<br>q = 0<br>doublet	D_0-D_1	446.59	0.0007	88A -> 89A 0.69835<br>81B -> 88B -0.25766<br>87B -> 88B -0.60097	0.858
	D_1-D_0	643.44	0.0216	88A -> 89A -0.94297<br>81B -> 88B -0.10268<br>87B -> 88B 0.24819	0.803

Table S11

Absorption and emission properties in anion doublet compounds with one PVA oligomer using TDA/ωB97XD/6-31G(d).

Species	Transition	λ, nm	f	Assignment	<S^2>
**A**<br>q = -1, doublet<br>One PVA chain<br>Optimized D_0<br>geometry	D_0-D_1	1334.67	0.0002	104A ->105A 0.98054<br>104A ->112A -0.12823	0.798
	D_0-D_2	563.16	0.1552	104A ->106A 0.95314<br>102B ->104B 0.13473<br>103B ->105B -0.21230	0.802
**A**<br>q = -1, doublet<br>One PVA chain<br>Optimized D_2<br>geometry	D_1-D_0	2553.80	0.0010	104A ->105A 0.98331<br>104A ->111A 0.12591	0.795
	D_2-D_0	678.75	0.1764	104A ->106A 0.96154<br>102B ->104B 0.15022<br>103B ->105B -0.15751	0.805
**B**<br>q = -1, doublet<br>One PVA chain<br>Optimized D_0<br>geometry	D_0-D_1	1550.33	0.0007	116A ->117A 0.98172	0.797
	D_0-D_2	588.20	0.1334	116A ->118A 0.95528<br>114B ->116B 0.14516<br>115B ->117B -0.19821	0.807
**B**<br>q = -1, doublet<br>One PVA chain<br>Optimized D_2<br>geometry	D_1-D_0	3904.98	0.0023	116A ->117A -0.98101	0.790
	D_2-D_0	720.01	0.1325	116A ->118A 0.95595<br>114B ->117B 0.11077<br>115B ->116B 0.18775	0.834
**C**<br>q = -1, doublet<br>One PVA chain<br>Optimized D_0<br>geometry	D_0-D_1	1423.60	0.0009	140A ->141A 0.97855	0.797
	D_0-D_2	583.27	0.1554	140A ->142A 0.95344<br>138B ->140B -0.13607<br>139B ->141B -0.20084	0.802
**C**<br>q = -1, doublet<br>One PVA chain<br>Optimized D_2<br>geometry	D_1-D_0	3179.42	0.0015	140A ->141A -0.98346<br>140A ->153A 0.10466	0.794
	D_2-D_0	716.92	0.1665	140A ->142A 0.96281<br>138B ->140B 0.14522<br>139B ->141B -0.13400	0.812
**D**<br>q = -1, doublet<br>One PVA chain<br>Optimized D_0<br>geometry	D_0-D_1	1350.46	0.0002	92A -> 93A 0.97717<br>92A -> 97A -0.12799	0.808
	D_0-D_2	638.13	0.1097	92A -> 94A 0.95866<br>89B -> 92B -0.12276<br>91B -> 93B 0.16537	0.805
**D**<br>q = -1, doublet<br>One PVA chain<br>Optimized D_2<br>geometry	D_1-D_0	2718.17	0.0011	92A -> 93A 0.98023<br>92A -> 97A 0.12788	0.801
	D_2-D_0	810.21	0.1225	92A -> 94A 0.96544<br>89B -> 92B 0.14133<br>91B -> 93B 0.10159	0.803
**E**<br>q = -1, doublet<br>One PVA chain<br>Optimized D_0<br>geometry	D_0-D_1	1244.05	0.0000	80A -> 81A 0.97688<br>80A -> 84A 0.11814	0.817
	D_0-D_2	600.48	0.0990	80A -> 82A 0.95219<br>79B -> 81B -0.19425	0.810
**E**<br>q = -1, doublet<br>One PVA chain<br>Optimized D_2<br>geometry	D_1-D_0	2065.06	0.0008	80A -> 81A 0.97937<br>80A -> 84A 0.11719	0.809
	D_2-D_0	736.67	0.1170	80A -> 82A 0.95925<br>77B -> 80B 0.14438<br>79B -> 81B -0.12868	0.809

**Ph-1COOH**<br>q = -1, doublet<br>One PVA chain<br>Optimized D_0<br>geometry	D_0-D_1	546.5	0.0049	70A -> 71A 0.98126<br>68B -> 70B -0.11612	0.806
	D_0-D_2	345.95	0.0918	70A -> 72A 0.84914<br>69B -> 70B 0.49965	0.788
**Ph-1COOH**<br>q = -1, doublet<br>One PVA chain<br>Optimized D_2<br>geometry	D_1-D_0	1876.82	0.0012	70A -> 71A -0.98899	0.784
	D_2-D_0	312.88	0.0001	70A -> 73A -0.58241<br>70A -> 74A -0.18301<br>70A -> 75A 0.75387	0.813
**Ph-2COOH**<br>q = -1, doublet<br>One PVA chain<br>Optimized D_0<br>geometry	D_0-D_1	1044.96	0.0253	81A -> 82A 0.95442<br>81A -> 83A 0.25134	0.808
	D_0-D_2	402.31	0.1005	81A -> 83A 0.89659<br>79B -> 81B 0.20845<br>80B -> 82B -0.27292	0.892
**Ph-2COOH**<br>q = -1, doublet<br>One PVA chain<br>Optimized D_2<br>geometry	D_1-D_0	1563.81	0.0154	81A -> 82A 0.94031<br>81A -> 83A 0.30744	0.793
	D_2-D_0	491.44	0.1380	81A -> 82A -0.28085<br>81A -> 83A 0.90521<br>80B -> 82B -0.22266	0.837
**Ph-3COOH**<br>q = -1, doublet<br>One PVA chain<br>Optimized D_0<br>geometry	D_0-D_1	1253.23	0.0003	92A -> 93A 0.98143	0.793
	D_0-D_2	515.98	0.1457	92A -> 94A 0.94568<br>90B -> 93B 0.14803<br>91B -> 92B -0.22472	0.812
**Ph-3COOH**<br>q = -1, doublet<br>One PVA chain<br>Optimized D_2<br>geometry	D_1-D_0	2655.82	0.0009	92A -> 93A 0.98404<br>92A ->102A -0.10664	0.791
	D_2-D_0	623.74	0.1617	92A -> 94A 0.95671<br>90B -> 92B -0.17181<br>91B -> 93B -0.16581	0.819
**Ph-4COOH**<br>q = -1, doublet<br>One PVA chain<br>Optimized D_0<br>geometry	D_0-D_1	669.18	0.0037	103A ->104A 0.95588<br>103A ->105A 0.18581<br>103A ->108A 0.15308	0.794
	D_0-D_2	458.81	0.2173	103A ->105A 0.91078<br>101B ->103B 0.21593<br>102B ->104B 0.20102	0.830
**Ph-4COOH**<br>q = -1, doublet<br>One PVA chain<br>Optimized D_2<br>geometry	D_1-D_0	778.56	0.0025	103A ->104A 0.97096<br>103A ->108A 0.14241	0.787
	D_2-D_0	503.15	0.2394	103A ->105A 0.93941<br>101B ->103B -0.21225<br>102B ->104B -0.20929	0.825
**Ph-5COOH**<br>q = -1, doublet<br>One PVA chain<br>Optimized D_0<br>geometry	D_0-D_1	741.02	0.0009	114A ->115A 0.97446<br>114A ->119A 0.12489	0.793
	D_0-D_2	445.79	0.1519	114A ->116A 0.91851<br>113B ->114B -0.23376<br>113B ->115B -0.18428	0.836
**Ph-5COOH**<br>q = -1, doublet<br>One PVA chain<br>Optimized D_2<br>geometry	D_1-D_0	878.45	0.0018	114A ->115A 0.97900<br>114A ->119A -0.12727	0.787
	D_2-D_0	500.73	0.1627	114A ->116A -0.93952<br>110B ->114B -0.17176<br>113B ->115B 0.22346	0.841
**Ph-6COOH**<br>q = -1, doublet<br>One PVA chain<br>Optimized D_0<br>geometry	D_0-D_1	662.49	0.0010	125A ->126A 0.97658<br>125A ->131A 0.12214	0.800
	D_0-D_2	446.84	0.1708	125A ->127A 0.91106<br>123B ->126B -0.12597<br>124B ->125B 0.32776	0.814
**Ph-6COOH**<br>q = -1, doublet<br>One PVA chain<br>Optimized D_2<br>geometry	D_1-D_0	922.46	0.0013	125A ->126A -0.98191<br>125A ->131A 0.10959	0.796
	D_2-D_0	520.68	0.2187	125A ->127A -0.94553<br>123B ->126B -0.15101<br>124B ->125B 0.19661	0.827

Table S12

Absorption and emission properties in hydrogenated doublet compounds with one PVA oligomer using TDA/ωB97XD/6-31G(d).

Species	Transition	λ, nm	f	Assignment	<S²>
**A**<br>q = 0, doublet<br>One PVA chain<br>Optimized D_0 geometry	D_0-D_1	447.70	0.0004	104A ->105A 0.69328<br>100B ->104B -0.67368	0.835
	D_0-D_2	341.58	0.0009	98B ->104B -0.21273<br>99B ->104B 0.83634<br>99B ->105B -0.29058	1.118
**A**<br>q = 0, doublet<br>One PVA chain<br>Optimized D_1 geometry	D_1-D_0	485.13	0.0001	104A ->105A -0.71152<br>101B ->104B 0.66555	0.813
	D_2-D_0	354.11	0.0815	104A ->105A -0.39522<br>99B ->104B 0.67906<br>101B ->104B -0.43594	1.042
**B**<br>q = 0, doublet<br>One PVA chain<br>Optimized D_0 geometry	D_0-D_1	448.86	0.0005	116A ->117A 0.68771<br>112B ->116B -0.66246<br>115B ->116B 0.11987	0.836
	D_0-D_2	344.50	0.0006	111B ->116B 0.86361<br>111B ->117B -0.30823<br>111B ->118B -0.21474	1.114
**B**<br>q = 0, doublet<br>One PVA chain<br>Optimized D_1 geometry	D_1-D_0	487.37	0.0001	116A ->117A 0.71302<br>112B ->116B -0.63405<br>113B ->116B -0.15218	0.814
	D_2-D_0	356.99	0.0918	116A ->117A -0.41284<br>111B ->116B 0.66433<br>112B ->116B -0.43150	1.023
**C**<br>q = 0, doublet<br>One PVA chain<br>Optimized D_0 geometry	D_0-D_1	446.76	0.0015	140A ->141A 0.67021<br>136B ->140B 0.69749	0.833
	D_0-D_2	351.16	0.0003	135B ->140B 0.85999<br>135B ->141B 0.30927<br>135B ->142B -0.20117	1.113
**C**<br>q = 0, doublet<br>One PVA chain<br>Optimized D_1 geometry	D_1-D_0	486.31	0.0019	140A ->141A 0.66528<br>137B ->140B 0.67056<br>138B ->140B -0.21474	0.812
	D_2-D_0	366.49	0.0232	135B ->140B 0.77757<br>135B ->141B 0.33798<br>137B ->140B -0.29511	1.111
**D**<br>q = 0, doublet<br>One PVA chain<br>Optimized D_0 geometry	D_0-D_1	486.34	0.0000	89B -> 93B 0.35734<br>90B -> 92B 0.45399<br>91B -> 92B 0.60289	1.268
	D_0-D_2	473.24	0.0003	89B -> 92B 0.71927<br>91B -> 92B -0.34057<br>91B -> 93B 0.26840	1.262
**D**<br>q = 0, doublet<br>One PVA chain<br>Optimized D_1 geometry	D_1-D_0	958.57	0.0000	90B -> 92B -0.25551<br>91B -> 92B -0.81141<br>91B -> 93B -0.39147	1.082
	D_2-D_0	498.77	0.0106	92A -> 93A 0.54916<br>87B -> 92B 0.65369<br>88B -> 92B -0.37017	0.917
**D**<br>q = 0, doublet<br>One PVA chain<br>Optimized D_2 geometry	D_1-D_0	1020.22	0.0000	91B -> 92B 0.89522<br>91B -> 94B -0.38315<br>91B -> 95B -0.14586	1.007
	D_2-D_0	441.96	0.0021	92A -> 93A 0.57894<br>85B -> 92B -0.45708<br>86B -> 92B -0.43868	1.021
**E**<br>q = 0, doublet<br>One PVA chain<br>Optimized D_0 geometry	D_0-D_1	508.24	0.0000	76B -> 80B 0.43088<br>76B -> 81B 0.30448<br>77B -> 80B 0.69178	1.316
	D_0-D_2	484.84	0.0000	76B -> 80B 0.69021<br>77B -> 80B -0.41813<br>77B -> 81B 0.26194	1.280
**E**<br>q = 0, doublet<br>One PVA chain<br>Optimized D_1 geometry	D_1-D_0	1008.88	0.0000	77B -> 80B -0.85349<br>77B -> 81B 0.40970<br>77B -> 82B -0.22143	1.024
	D_2-D_0	502.37	0.0157	80A -> 81A 0.51512<br>75B -> 80B 0.50841<br>76B -> 80B -0.60885	1.030
**E**<br>q = 0, doublet<br>One PVA chain	D_1-D_0	896.31	0.0000	77B -> 80B -0.84165<br>77B -> 81B 0.43510<br>77B -> 82B -0.21486	1.094

Optimized D_2 geometry	D_2-D_0	473.12	0.0001	73B -> 80B -0.29340<br>76B -> 80B -0.67618<br>76B -> 81B -0.39884	1.534
**Ph-1COOH**<br>q = 0, doublet<br>One PVA chain<br>Optimized D_0<br>geometry	D_0-D_1	405.48	0.0206	70A -> 72A 0.46070<br>67B -> 70B 0.84601<br>67B -> 72B -0.13407	0.827
	D_0-D_2	304.51	0.0015	63B -> 70B 0.60072<br>64B -> 70B -0.23446<br>65B -> 70B 0.57506	1.029
**Ph-1COOH**<br>q = 0, doublet<br>One PVA chain<br>Optimized D_1<br>geometry	D_1-D_0	437.85	0.0082	70A -> 71A -0.59802<br>67B -> 70B 0.77022<br>67B -> 72B -0.11733	0.813
	D_2-D_0	321.77	0.0002	64B -> 70B 0.74125<br>64B -> 72B 0.28222<br>65B -> 70B 0.52269	1.112
**Ph-2COOH**<br>q = 0, doublet<br>One PVA chain<br>Optimized D_0<br>geometry	D_0-D_1	431.28	0.0026	81A -> 82A -0.65458<br>81A -> 83A 0.15534<br>79B -> 81B 0.68654	0.834
	D_0-D_2	340.52	0.0001	76B -> 82B -0.20697<br>77B -> 81B 0.89109<br>77B -> 83B 0.23646	1.056
**Ph-2COOH**<br>q = 0, doublet<br>One PVA chain<br>Optimized D_1<br>geometry	D_1-D_0	469.44	0.0013	81A -> 82A -0.68239<br>79B -> 81B -0.52064<br>80B -> 81B 0.44782	0.810
	D_2-D_0	344.51	0.0932	81A -> 82A -0.47298<br>77B -> 81B -0.55901<br>79B -> 81B 0.36356	1.038
**Ph-3COOH**<br>q = 0, doublet<br>One PVA chain<br>Optimized D_0<br>geometry	D_0-D_1	443.22	0.0003	92A -> 93A 0.74954<br>87B -> 92B -0.47070<br>88B -> 92B -0.28905	0.844
	D_0-D_2	380.24	0.0015	86B -> 92B 0.28100<br>87B -> 92B -0.29688<br>88B -> 92B 0.82036	1.028
**Ph-3COOH**<br>q = 0, doublet<br>One PVA chain<br>Optimized D_1<br>geometry	D_1-D_0	744.50	0.0000	89B -> 92B -0.15658<br>90B -> 92B 0.90332<br>90B -> 94B 0.32067	0.958
	D_2-D_0	422.71	0.0585	87B -> 92B 0.50765<br>88B -> 92B 0.55562<br>89B -> 92B 0.42301	0.931
**Ph-4COOH**<br>q = 0, doublet<br>One PVA chain<br>Optimized D_0<br>geometry	D_0-D_1	429.12	0.0012	103A ->104A 0.65097<br>100B ->103B 0.38539<br>101B ->103B -0.53170	0.857
	D_0-D_2	348.76	0.0038	99B ->103B 0.63372<br>100B ->103B -0.43018<br>101B ->103B -0.47629	1.013
**Ph-4COOH**<br>q = 0, doublet<br>One PVA chain<br>Optimized D_1<br>geometry	D_1-D_0	542.9	0.0124	103A ->104A 0.91660<br>100B ->103B 0.26531<br>101B ->103B 0.13078	0.834
	D_2-D_0	340.26	0.0425	103A ->105A 0.29931<br>100B ->103B 0.67809<br>101B ->103B 0.28685	1.210
**Ph-5COOH**<br>q = 0, doublet<br>One PVA chain<br>Optimized D_0<br>geometry	D_0-D_1	471.73	0.0113	114A ->115A 0.83338<br>110B ->114B -0.34878<br>111B ->114B 0.31375	0.827
	D_0-D_2	327.22	0.1548	114A ->115A -0.49973<br>110B ->114B -0.56069<br>111B ->114B 0.58336	0.827
**Ph-5COOH**<br>q = 0, doublet<br>One PVA chain<br>Optimized D_1<br>geometry	D_1-D_0	620.64	0.0217	114A ->115A -0.94204<br>110B ->114B 0.12025<br>111B ->114B 0.22168	0.804
	D_2-D_0	344.43	0.0036	110A ->115A -0.41816<br>114A ->116A -0.48988<br>111B ->115B -0.45762	1.971
**Ph-6COOH**<br>q = 0, doublet<br>One PVA chain<br>Optimized D_0<br>geometry	D_0-D_1	449.36	0.0008	125A ->126A 0.72231<br>121B ->125B -0.15621<br>123B ->125B 0.56984	0.853
	D_0-D_2	347.74	0.0741	125A ->126A -0.49541<br>120B ->125B -0.27538<br>123B ->125B 0.59499	1.002

**Ph-6COOH**<br>q = 0, doublet<br>One PVA chain<br>Optimized D_1<br>geometry	D_1-D_0	586.01	0.0233	125A ->126A -0.92745<br>123B ->125B -0.26100	0.814
	D_2-D_0	356.75	0.0371	125A ->127A -0.30776<br>123B ->125B -0.66298<br>123B ->126B 0.33410	1.542

Tabel S13 Simualtions of compound E with CASPT2 method.

		E R= -COH		
		anion	cation	neutral
D_1/S_1	eV	0.32	0.31	2.61
D_2/S_2		1.36	0.41	3.75
Q_1		3.28	3.01	
Q_2		3.50	3.05	
$D_1 \rightarrow D_0$	X	0.41	-0.05	0.01
	Y	0.12	-0.01	-0.03
	Z	-0.32	0.02	0.002
	Osc.	0.002	2.7e-5	5e-5
$D_2 \rightarrow D_0$	X	1.42	-0.05	0.036
	Y	0.54	-0.02	0.043
	Z	0.37	0.03	0.39
	Osc	0.082	4.1E-5	0.015
$D_2 \rightarrow D_1$	X	-0.38	-0.01	-8E-4
	Y	-0.03	-0.01	8.5E-4
	Z	1.27	-0.16	9.4E-4
	Osc.	0.0451	5.7E-5	0.0
$k_R(D_1 \rightarrow D_0)$, s^{-1}		8781	123	14605
$k_R(D_2 \rightarrow D_1)$, s^{-1}		2087055	26	0
$k_R(D_2 \rightarrow D_0)$, s^{-1}		6503489	288	9045000.0
$k_{IC}(D_1 \rightarrow D_0)$, s^{-1}		5.788e+11	2.163e+12	1.536e+05
$k_{IC}(D_2 \rightarrow D_1)$, s^{-1}		3.356e+07	1.491e+11	5.466e+12
$k_{IC}(D_2 \rightarrow D_0)$, s^{-1}		2.917e+09	1.263e+12	1.166e+05
$\varphi(D_1 \rightarrow D_0)$		1.52e-08	5.7e-11	0.087
$\varphi(D_2 \rightarrow D_1)$		7.1e-4	1.8e-11	0
$\varphi(D_2 \rightarrow D_0)$		2.2e-3	2e-10	1.65e-6

Tabel S14 Simualtions of compound B with CASPT2 method.

		B R= -COOH		
		anion	cation	neutral
		CASPT2	CASPT2	CASPT2
D_1/S_1	eV	0.32	0.06	4.16
D_2/S_2		1.91	0.11	4.97
Q_1		3.45	3.13	
Q_2		3.60	3.16	
$D_1 \rightarrow D_0$	X	0.236	-0.008	0.23
	Y	0.031	0.38	0.52
	Z	5.0E-5	0.002	2.1E-5
	Osc.	7e-4	2e-4	0.034
$D_2 \rightarrow D_0$	X	-0.206	0.002	0.052
	Y	1.07	0.002	1.24
	Z	-4E-5	-8E-4	-1.9E-5
	Osc	0.056	0.0	0.18
$D_2 \rightarrow D_1$	X	-1.22	-0.044	0.011
	Y	-0.25	0.18	0.42
	Z	-1.9E-4	0.002	-6.0E-6
	Osc.	0.06	4.6E-5	0.003
$k_R(D_1 \rightarrow D_0)$, s^{-1}		3073	61	25230180
$k_R(D_2 \rightarrow D_1)$, s^{-1}		6586368	25	82329.6
$k_R(D_2 \rightarrow D_0)$, s^{-1}		8760109	0	190651426
$k_{IC}(D_1 \rightarrow D_0)$, s^{-1}		7.762e+12	3e+12	1.816e+03
$k_{IC}(D_2 \rightarrow D_1)$, s^{-1}		8.749e+08	2e+11	3.122e+10
$k_{IC}(D_2 \rightarrow D_0)$, s^{-1}		2.380e+09	1.8e+12	3.398e+02
$\varphi(D_1 \rightarrow D_0)$		4e-10	2e-11	0.99
$\varphi(D_2 \rightarrow D_1)$		2e-3	1e-11	2.6e-6
$\varphi(D_2 \rightarrow D_0)$		2.6e-3	0	6.1e-3

Tabel S15 Radiative and non-radiative rate constants, simualted on theoretically obtained transition energies $D_2 \rightarrow D_0$, $D_1 \rightarrow D_0$ for studied compounds in both emission mechanisms.

	D_1	D_2	$D_1 \rightarrow D_0$	$D_2 \rightarrow D_0$	$k_R(D_1 \rightarrow D_0)$	$k_R(D_2 \rightarrow D_0)$	$k_{IC}(D_1 \rightarrow D_0)$	$k_{IC}(D_2 \rightarrow D_0)$	QY
	eV	eV	f	f	s^{-1}	s^{-1}	s^{-1}	s^{-1}	
A^-	0.22	1.72	0.0013	0.13	2.7E+03	1.7E+07	3.1E+14	4.6E+09	3.68E-03
AH	2.57		0.0001		2.8E+04		4.6E+05		5.74E-02
B^-	0.22	1.70	0.0014	0.14	2.9E+03	1.7E+07	6.7E+14	1.2E+10	1.41E-03
BH	2.57		0.0001		2.8E+04		3.2E+05		8.05E-02
C^-	0.23	1.67	0.0017	0.15	3.8E+03	1.8E+07	3.0E+14	7.7E+09	2.33E-03
CH	2.57		0		0		9.6E+05		0
D^-	0.23	1.39	0.0017	0.11	3.7E+03	9.4E+06	2.5E+14	2.0E+10	4.7E-04
DH	1.36		0		0		2.0E+09		0
E^-	0.24	1.46	0.0015	0.11	3.6E+03	9.8E+06	1.2E+14	1.3E+10	7.53E-04
EH	1.35		0		0		6.0E+08		0
Ph-1R^-	0.80	3.33	0.0009	0.20	2.4E+04	9.7E+07	9.3E+11	4.3E+06	9.58E-01
Ph-1RH	2.87		0.0082		2.9E+06		1.3E+07		1.82E-01
Ph-2R^-	0.78	2.64	0.0230	0.15	6.0E+05	4.4E+07	2.0E+11	7.7E+07	3.64E-01
Ph-2RH	2.70		0.0019		5.9E+05		1.3		1
Ph-3R^-	0.23	1.78	0.0010	0.12	2.2E+03	1.6E+07	1.4E+14	2.0E+09	7.94E-03
Ph-3RH	2.57		0.0005		1.4E+05		4.8E+05		2.26E-01
Ph-4R^-	2.51	1.08	0.2334	0.00	6.3E+07	1.5E+04	1.6E+10	2.6E+07	5.77E-04
Ph-4RH	1.62		0.0009		1.0E+05		3.6E+08		2.78E-04
Ph-5R^-	0.40	1.98	0.0007	0.13	4.7E+03	2.2E+07	7.7E+10	3.5E+07	3.86E-01
Ph-5RH	1.90		0.019		2.9E+06		1.1E+07		2.09E-01
Ph-6R^-	1.33	2.50	0	0.18	0	4.8E+07	1.1E+08	1.6E+06	9.68E-01
Ph-6RH	1.93		0.0216		3.4E+06		2.3E+06		5.96E-01
R=COOH									

Tabel S16 Conversion of experimentally recorded emission wavelengths to energy for studied compounds.

	λ_{exp}	$Energy, exp$
	nm	eV
A^-	560	2.214
AH	560	2.214
B^-	560	2.214
BH	560	2.214
C^-	360	3.444
CH	360	3.444
D^-	600	2.066
DH	600	2.066
E^-	575	2.156
EH	575	2.156
Ph-1R^-	403	3.077
Ph-1RH	403	3.077
Ph-2R^-	510	2.431
Ph-2RH	510	2.431
Ph-3R^-	571	2.171
Ph-3RH	571	2.171
Ph-4R^-	450	2.755
Ph-4RH	450	2.755
Ph-5R^-	557	2.226
Ph-5RH	557	2.226
Ph-6R^-	419	2.959
Ph-6RH	419	2.959
R=COOH		

Tablel S17 Radiative and non-radiative rate constants, simualted on experimentally measured emission wavelengthes (transition energies) for studied compounds.

	D_1	D_2	$D_1 \rightarrow D_0$	$D_2 \rightarrow D_0$	$k_R(D_1 D_0)$	$k_R(D_2 D_0)$	$k_{IC}(D_1 D_0)$	$k_{IC}(D_2 D_0)$	$k_{IC}(D_2 D_1)$	QY
	eV	eV	f	f	s^{-1}	s^{-1}	s^{-1}	s^{-1}	s^{-1}	
A^-	0.22	2.21	0.0013	0.13	2.7E+03	2.8E+07	3.08E+14	1.9E+08	4.3E+07	0.11
B^-	0.22	2.21	0.0014	0.14	2.9E+03	2.9E+07	6.66E+14	4.4E+08	1.4E+08	0.05
C^-										
D^-	0.23	2.07	0.0017	0.11	3.7E+03	2.1E+07	2.51E+14	2.3E+08	4.3E+07	0.07
E^-	0.24	2.16	0.0015	0.11	3.6E+03	2.1E+07	1.18E+14	1.5E+08	1.7E+07	0.11
Ph-1R$^-$	0.80	3.08	0.0009	0.20	2.4E+04	8.3E+07	9.32E+11	5.6E+06	1.8E+06	0.92
Ph-2R$^-$	0.78	2.43	0.0230	0.15	6.0E+05	3.7E+07	2.02E+11	1.0E+08	1.1E+08	0.15
Ph-3R$^-$	0.23	2.17	0.0010	0.12	2.2E+03	2.4E+07	1.43E+14	1.3E+08	2.9E+07	0.13
Ph-4R$^-$	2.51	2.75	0.2334	0	6.3E+07	9.7E+04	1.62E+10	4.6E+06	1.9E+07	0.73
Ph-5R$^-$	0.40	2.23	0.0007	0.13	4.7E+03	2.8E+07	7.68E+10	4.9E+06	1.0E+04	0.85
Ph-6R$^-$	1.33	2.96	0	0.18	0	6.7E+07	1.11E+08	1.1E+05	1.5E+04	1.00
AH	2.21		0.0001		2.1E+04		1.4E+06			0.02
BH	2.21		0.0001		2.1E+04		9.5E+05			0.02
CH										
DH	2.07		0		0		4.9E+07			0
EH	2.16		0		0		9.3E+06			0
Ph-1RH	3.08		0.0082		3.3E+06		2.7E+06			0.55
Ph-2RH	2.43		0.0019		4.8E+05		2.5E+07			0.02
Ph-3RH	2.17		0.0005		1.0E+05		1.7E+06			0.06
Ph-4RH	2.75		0.0009		2.9E+05		1.5E+06			0.17
Ph-5RH	2.23		0.019		4.0E+06		1.2E+06			0.77
Ph-6RH	2.96		0.0216		8.1E+06		1.1E+04			1
R=COOH										
